# The mediating role of epigenetic ageing in the nonlinear association between body mass index and survival: a prospective cohort analysis of the US Health and Retirement Study

**DOI:** 10.1016/j.ebiom.2025.105883

**Published:** 2025-08-16

**Authors:** Peggy Ler, Juulia Jylhävä, Sara Hägg, Deborah Finkel, Anna K. Dahl Aslan, Alexander Ploner, Ida K. Karlsson

**Affiliations:** a Department of Medical Epidemiology and Biostatistics, Karolinska Institutet, Stockholm, Sweden; b Faculty of Medicine and Health Technology, and Gerontology Research Center, University of Tampere, Tampere, Finland; c Tampere Institute for Advanced Study, Tampere University, Tampere, Finland; d Center for Economic and Social Research, University of Southern California, Los Angeles, CA, USA; e Institute for Gerontology, Jönköping University, Jönköping, Sweden; f School of Health Sciences, University of Skövde, Skövde, Sweden

**Keywords:** Biological age, Body mass index, Epigenetic age, Mediation analysis, Mortality, Obesity

## Abstract

**Background:**

The role of biological ageing in the association between body mass index (BMI) and survival remains unclear. We examined whether epigenetic age acceleration (EAA), a biomarker of biological ageing, mediates the BMI-survival association.

**Methods:**

We analysed data from 3840 participants (aged 51–100) in the 2016 US Health and Retirement Study, with survival information through 2020. Mediation analyses were performed using linear regression and Gompertz proportional hazards models with restricted cubic splines, adjusting for age, sex, ethnicity/race, smoking, education, and metabolic health. Average direct effects (ADE) of BMI and average causal mediation effects of EAA (HannumAgeAcc, PhenoAgeAcc, GrimAgeAcc, and DunedinPace) on survival time were estimated with 95% confidence intervals (CI).

**Findings:**

Associations between BMI, EAA, and survival were nonlinear: high and low BMIs were associated with higher EAA and reduced survival time. ADEs of high BMI (35 kg/m^2^ versus 27 kg/m^2^) were not statistically significant (reduced survival time: 1.21–1.58 years) but significant for low BMI (19 kg/m^2^ versus 27 kg/m^2^, reduced survival time: 5.60–6.38 years). For high BMI, mediation was significant through all EAAs, with reduced survival time ranging from 0.28 to 0.71 years, accounting for 15–37% of total effects. For low BMI, mediation was statistically significant through HannumAgeAcc (reduced survival time: 0.44, CI: 0.08–0.86) and GrimAgeAcc (reduced survival time: 0.73, CI: 0.15–1.38), accounting for 7–11% of total effects.

**Interpretation:**

EAA partially mediated the high BMI-survival association, supporting the mediating role of accelerated ageing in the obesity-survival relationship. Mediation through EAA in the low BMI-survival association was weaker, indicating that alternative mechanisms, other than accelerated ageing, may dominate.

**Funding:**

10.13039/501100006636Forte, Vetenskaprådet, SFOepi, Karolinska Institutet's Research Foundation, Loo and Hans Osterman Foundation, the Foundation for Geriatric Diseases at Karolinska Institutet.

**Research in context**

**Evidence before this study**

We searched PubMed for studies using the terms (“obesity” OR “BMI” OR “body mass index”) AND (“biological age” OR “epigenetic age” OR “epigenetic clock”) from database inception to May 2025, without language restrictions. The research that examined the association between body mass index and epigenetic ageing reported mixed findings: some studies found significant associations between BMI and epigenetic age only in specific tissues, and one of the evaluated age-specific associations reported significant associations in middle-aged but not older adults. None has evaluated the potential nonlinear association between BMI and epigenetic age, nor the potential mediating role of epigenetic age in the BMI-survival relationship.

**Added value of this study**

Our study carefully examined the mediating role of epigenetic age in the association between BMI and survival, which has been well-established as nonlinear in previous studies. We found that the association between BMI and epigenetic ageing was also nonlinear, shedding light on the mediating role of epigenetic ageing in the association between both low and high BMIs and survival. We found significant mediation effects of epigenetic ageing in the relationship between high BMI and survival, substantiating the hypothesis that obesity accelerates biological ageing, thereby reducing survival. In contrast, in the association between low BMI and survival, we found less evidence of mediation through epigenetic ageing but stronger direct effects of low BMI on survival, highlighting that the mechanisms linking high BMI and low BMI to survival are potentially distinct.

**Implications of all the available evidence**

Our findings support the hypothesis that high BMI accelerates ageing, thereby reducing survival, underscoring crucial public health implications in the context of rising obesity prevalence, a rapidly ageing population, and an increasing health burden from age-related illnesses that may ensue. Future research should aim to disentangle the biological mechanisms that connect higher or lower BMI, biological ageing, and lifespan.

## Introduction

Obesity and ageing are multifaceted conditions that share similarities in pathophysiological mechanisms and contribute to age-related diseases and mortality.^1^^,^^2^ Obesity, defined as the excessive accumulation of adipose tissue, is commonly characterised by a body mass index (BMI) of 30 kg/m^2^ or higher.^3^ Ageing is characterised by a progressive loss of the body's structural integrity and physiological functions.^2^ The commonalities between obesity and ageing suggest that interventions addressing one condition may impact the other, improving overall health and life expectancy.^2^ Targeting obesity through lifestyle changes and weight management, as well as metabolic health, may potentially slow biological ageing, which could in turn extend healthspan and lifespan. Understanding the relationship between obesity, ageing, and life expectancy is therefore critical, particularly in our current global context, where the prevalence of obesity and the population of older adults are growing.

Biological ageing varies among individuals, and quantifying ageing solely by chronological years fails to capture these variations between individuals, which may occur at the molecular, physiological, and functional levels.^4^ Epigenetic clocks address this inter-individual heterogeneity in ageing by measuring molecular-level epigenetic changes, specifically methylation of CpG dinucleotides (CpGs) at specific genome sites.^4^ Since their development, epigenetic clocks have evolved from accurate estimators of chronological age to predictors of age-related phenotypes, morbidity, and mortality, highlighting their ability to distil fundamental aspects of complex biological ageing processes into a single, interpretable measure.^4^ A predicted epigenetic age higher or lower than chronological age suggests an acceleration or a deceleration in biological ageing.^5^ One particular epigenetic clock, DunedinPACE, estimates the pace of ageing, indicating how quickly an individual ages relative to their chronological age.^6^ These epigenetic clocks measure biological ageing and late-life health, enriching our understanding of biological ageing and its relationship with other phenotypes.^4^

Given the pathophysiological similarities between obesity and ageing, obesity is hypothesised to accelerate biological ageing.^1^^,^^2^ However, the association between obesity and ageing is complex, with studies on the association between high BMI and epigenetic ageing yielding mixed findings.^7^^,^^8^ Some research has suggested that the impact of high BMI on epigenetic ageing may be tissue-specific^7^ and age-specific, with associations found primarily in middle-aged but not older adults.^9^ While the findings are inconsistent, no study has examined epigenetic ageing as a mediator in the BMI-survival relationship, a critical gap this study aims to address.

Prior studies have predominantly used BMI as a linear measure, where higher BMI was associated with higher epigenetic age.^7^^,^^8^ Nevertheless, nonlinear relationships between BMI and health or survival have often been reported, where both low and high BMI were associated with adverse health and mortality risk.^10^, ^11^, ^12^ Different BMI levels may influence health through distinct mechanisms: a low BMI often reflects unintentional weight loss due to pre-existing illnesses, which increases mortality risk^13^^,^^14^; whereas a high BMI may contribute to mortality risk through its association with cardiovascular disease and type II diabetes.^3^ The nonlinear association between BMI and survival, the potential different mechanisms that link lower and higher BMI to survival, and the lack of evaluation of the nonlinear relationship between BMI and epigenetic age warrant the need to adopt nonlinear approaches to study this relationship.

Despite the hypothesis that obesity accelerates biological ageing^1^^,^^2^ and strong evidence indicating both low and high BMI as risk factors for mortality,^10^, ^11^, ^12^ no studies have explored the role of biological ageing in the relationship between BMI and mortality, with considerations of potential nonlinear associations between BMI and biological ageing and survival. This study, therefore, aimed to elucidate whether accelerated biological ageing explains the increased mortality risks associated with low and high BMI. We used epigenetic age acceleration (EAA) as a proxy measure of biological ageing. By including EAA derived from different epigenetic clocks, we aimed to examine the role of chronological age, physiological state, and ageing pace in the BMI-survival relationship. Given the potential for differing mechanisms linking low and high BMI with survival, we hypothesised that the mediation effect through EAA would be higher when the exposure was high BMI rather than low BMI.

## Methods

### Study population

The Health and Retirement Study (HRS) is a longitudinal survey of adults aged 50 years and above in the United States. It collects demographic, psychosocial, and health-related data through biennial follow-ups via face-to-face or phone interviews since 1992.^15^ All participants in HRS provided informed consent, and ethical approvals for data collection were granted by the Institutional Review Board at the University of Michigan. The Swedish Ethical Review Authority (2024-03706-0) approved this current study.

The HRS sampling adopted a multi-stage area probability design, which included geographic stratification, clustering, and oversampling for specific demographic groups.^15^ From 2006, data collection was enhanced to include physical measures for half the sample, whereas the other half underwent interviews, as in previous waves. We drew data from the 2016 Venous Blood Study, which collected venous blood and assessed fasting status during a home visit conducted within four weeks of the interview. While the response rate for the data collection (core and enhanced interviews) in 2016 was 73.9%,^16^ the consent rates for the Venous Blood Study were 78.5%, of which 82.9% underwent blood collection.^17^

The Venous Blood Study comprised 9934 respondents who consented to participate.^17^ A random selection of 4104 venous blood samples underwent DNA methylation assays, with 4018 samples passing quality control and being included in this current study.^17^

### Exposure—body mass index

We used self-reported height and weight from 2016, with height typically asked during the first interview. BMI was calculated as weight in kilograms divided by the square of height in metres. Height used in the calculation of BMI was cleaned, as described in detail previously.^18^ Briefly, the data cleaning involved examining the height recorded across waves for each individual and setting those with a standard deviation greater than one from the individual's mean to missing. This was followed by calculating the mean reported height for each individual, which was then used to calculate the BMI.

### Mediator–epigenetic age acceleration

We used the epigenetic age computed by HRS for this study. HRS used Infinium Methylation EPIC BeadChip (Illumina Inc., San Diego, CA, USA) to measure DNA methylation levels and compute several epigenetic clock measures.^19^ Epigenetic age derived from six epigenetic clocks—Horvath-I,^20^ Horvath-II,^4^ Hannum,^21^ PhenoAge,^22^ GrimAge,^23^ and DunedinPACE were used.^6^ These clocks measure epigenetic age by assessing DNA methylation levels at CpGs selected for their capacity to predict chronological age, phenotypes, and mortality risk.

Horvath-I was trained to predict chronological age using 353 CpGs from 51 healthy tissues and cell types.^20^ Horvath-II was designed to enhance chronological age prediction across multiple tissues, including fibroblasts and skin cells, by utilising 391 CpGs, thus reducing the influence of blood cell type counts.^4^ Hannum, based on 71 CpGs in leukocytes from whole blood, was also designed to measure chronological age.^21^

PhenoAge^22^ and GrimAge^23^ estimate epigenetic age using CpGs associated with chronological age, health phenotypes, and mortality risk. PhenoAge incorporates 513 CpGs linked with chronological age, clinical biomarkers (e.g., albumin, creatinine, glucose, and C-reactive protein), morbidities (e.g., cancers, Alzheimer's disease), and all-cause mortality.^22^ GrimAge uses 1030 CpGs associated with chronological age, smoking pack-years, sex, 12 plasma protein biomarkers of physiological health, and all-cause mortality.^23^ DunedinPACE estimates the rate of ageing, defined as a physiological change per calendar year, by averaging the rate of change across 19 biomarkers.^6^

EAA was calculated as residuals from regressing chronological age on the respective epigenetic clocks, yielding age acceleration for Horvath-I (HorvathAgeAcc-I), Horvath-II (HorvathAgeAcc-II), Hannum (HannumAgeAcc), PhenoAge (PhenoAgeAcc), and GrimAge (GrimAgeAcc). Positive EAA indicates accelerated ageing, while negative EAA suggests decelerated ageing. Since DunedinPACE measures the rate of ageing, age acceleration was not calculated.

### Outcome—all-cause mortality

HRS tracked respondents' vital status through interviews with respondents, their partners, or someone familiar with them.^15^ If a precise date of death was unavailable, it was imputed using the last known alive date, and the date of death status was confirmed.^15^

### Covariates

We included metabolic health status in the main models to estimate the associations between BMI and EAA or mortality that are not explained by obesity-related metabolic dysfunction. Adjusting for these variables likely yields more conservative estimates by excluding potential effects mediated through metabolic health. Metabolic health was determined from biomarkers assayed from the 2016 Venous Blood Study, based on a modified version of the National Cholesterol Education Program ATP III criteria for metabolic syndrome.^24^ Individuals were classified as metabolically healthy if they had none and metabolically unhealthy if they had at least one of the following: hypertension, hyperglycaemia, hypertriglyceridaemia, and low high-density lipoprotein cholesterol (HDL-C), using a combination of self-reported data and venous blood sampling results (Appendix p 3).

We also accounted for sociodemographic [age, sex, educational attainment, ethnicity or race (ethnicity/race)] and lifestyle factors (smoking status). Participants self-reported ethnicity/race as White/Caucasian, Black/African American, or other. Educational attainment was dichotomised into college/university education or higher versus below college/university education. Smoking status was classified as never, former, or current smokers.

### Statistical analysis

Respondents were followed from the age at which they were interviewed in 2016 until death or December 2020, whichever came first. Participants' characteristics in 2016 are presented as means (standard deviation) for continuous variables and counts (percentages) for categorical variables. We excluded participants with height <1.35 m (n = 3), BMI >60 kg/m^2^ (n = 5), and large variations in height across waves (n = 92, see Appendix p 4). We excluded 2.0% of individuals with missing data on: more than one metabolic health biomarker (n = 9), smoking status (n = 23), weight (n = 31), height (n = 14), and vital status (n = 1). To assess the potential impact of missing data, we compared individuals with and without missing values across the primary study variables; no differences were observed (Appendix p 5).

**Fig. 1:**
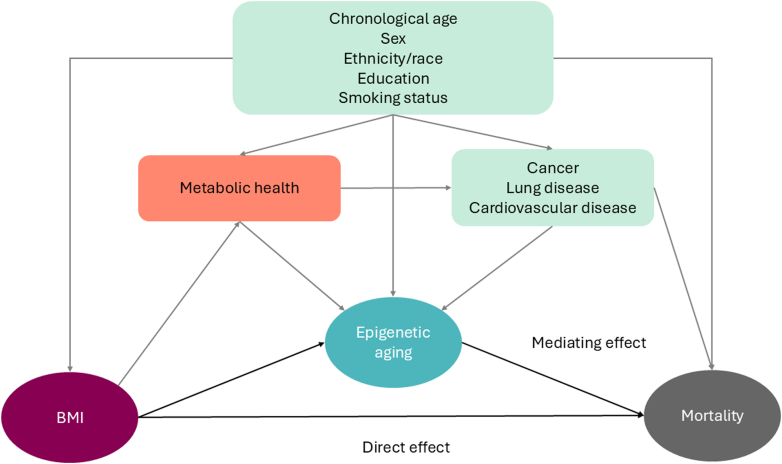
The directed acyclic graph (DAG) represents the primary hypothesis, which postulated that epigenetic ageing mediates the association between BMI and mortality risk. The DAG illustrates chronological age, sex, ethnicity/race, education, and smoking status as confounders that affect exposure (BMI), the mediator (epigenetic ageing), and outcome (all-cause mortality). Metabolic health and history of chronic diseases is shown in the DAG as potential mediators to association between BMI, explicitly high BMI, and mortality. Abbreviations: BMI—body mass index.

Fig. 1 illustrates the directed acyclic graph guiding the statistical analyses. We used linear regression to model the relationship between BMI and each EAA. To model the association of BMI and each EAA with survival, we applied Gompertz proportional hazard models with age as the underlying timescale, because Gompertz yielded the lowest Akaike information criterion compared to other distributions (Appendix p 6). Participants were right-censored at the end of the follow-up if they were still alive. Individuals with a confirmed death status were included in the survival analyses. We performed linear regression and Gompertz proportional hazards models with and without weights to examine the effects of weighted models.

Mediation analyses were conducted within a potential outcomes framework by Imai et al., which quantifies the direct and indirect (mediated) effects as the average difference in predicted outcome (marginal mean difference) between counterfactual scenarios: exposed versus unexposed.^25^

To estimate direct effects, the exposure variable (BMI) was set to exposed or unexposed levels while keeping the mediator (EAA) fixed at a constant level as predicted by the mediation model, with all other covariates left at their observed values. To estimate indirect (mediated) effects, the mediator variable was set to the value predicted by the mediation model for exposed or unexposed levels of the exposure variable, while keeping the exposure variable constant, with covariates held at their observed values. We termed the estimated effects average direct effects (ADE) and average causal mediation effects (ACME). When ADE and ACME are aligned (in the same direction), they can be expressed as a percentage of the total effect (ADE + ACME).

**Fig. 2:**
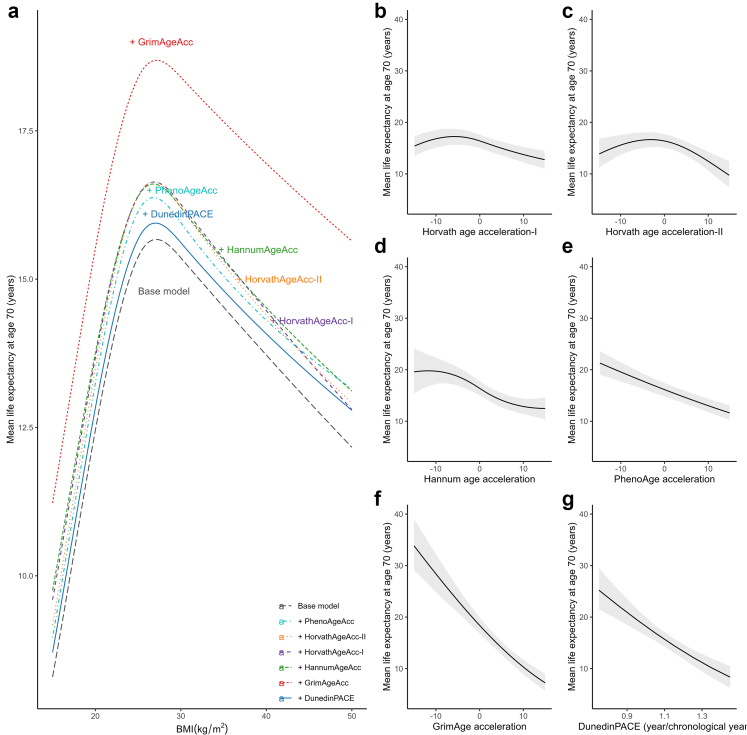
Association between body mass index (Panel a) and each epigenetic age acceleration measure (Panel b–g) with mean life expectancy (n = 3840). Panel a specifically shows the mean predicted life expectancy by BMI in a base model where the epigenetic age acceleration measure was not included and models adjusted for each epigenetic age acceleration measure. Mean life expectancies were estimated from the Gompertz proportional hazards model with age as the underlying time scale. All models were adjusted for age, sex, ethnicity/race, smoking status, educational attainment, and metabolic health. The base model does not include epigenetic age acceleration measures. The plots were based on predictions made among white males aged 70 years, with mean levels of epigenetic age acceleration measure or BMI, who were never smokers, metabolically unhealthy, and had education attainment of high school or below. Abbreviations: BMI—body mass index.

We performed separate mediation analyses for each EAA measure as a mediator of the effects of low and high BMI on mean survival time as the outcome. Low and high BMI were defined as an exposed level of 19 kg/m^2^ and 35 kg/m^2^, respectively, with 27 kg/m^2^ as the reference. The reference level of 27 kg/m^2^ was selected because it corresponded with the longest predicted survival time, based on findings from Gompertz proportional hazard models (see Results, Fig. 2). The equations for estimating ADE and ACME are presented in the supplement (Appendix p 7).

Using linear models for each EAA measure as mediation models and Gompertz proportional hazards models as the outcome models, the key outcomes reported—ADE and ACME—estimated the differences in predicted survival time between the exposed and unexposed scenarios in years. All models were adjusted for age, sex, ethnicity/race, smoking status, education, and metabolic health. Restricted cubic splines were used to capture nonlinear associations.

### Sensitivity analyses

Since there is no widely accepted definition of metabolic health, we conducted sensitivity analyses by repeating all analyses with a less strict definition, where metabolic health status was defined as having no more than one of the four metabolic components: hypertension, hyperglycaemia, hypertriglyceridaemia, and low HDL-C. Appendix (p 3) details the definitions of metabolic health.

To interpret ACME causally, four assumptions must be fulfilled: one, there is no unmeasured confounding between BMI and survival.^26^ Two, there is no unmeasured confounding between EAA and survival. Three, there is no unmeasured confounding between BMI and EAA. Four, no confounder in the EAA and survival relationship that is influenced by BMI. While residual confounding cannot be entirely ruled out, we conducted a series of sensitivity analyses to strengthen our findings. We repeated the mediation analyses, further adjusting for the history of cancer, lung disease, or cardiovascular disease obtained during telephone or in-person interviews in 2016. Including the history of chronic diseases in the sensitivity analyses allows us to estimate the effects not accounted for by obesity-related chronic diseases.

Next, we adjusted the mediation models for unhealthy alcohol use, defined as the self-reported consumption of more than two drinks in females and three drinks in males on any day in the past three months.

We repeated the mediation analysis using self-reported BMI data collected in 2014 to establish the temporal order of exposure and mediator. However, metabolic healthy status in 2014 was defined as the absence of hypertension, hyperglycemia, and low HDL-C (from dried blood spot measures or self-reported high cholesterol) because venous blood measures of triglyceride levels were unavailable and HDL-C measures were limited. Lastly, sex differences were assessed by stratifying the mediation analyses by sex.

All results are reported with 95% confidence intervals (CI). A modified version of the R package mediation (4.5.0) was used for mediation analyses.^27^ We reported bootstrap 95% CI for our estimands using 3000 resamples. All analyses were conducted in R v4.2.3.^28^

Funders of this research do not play any role in the study design, data analyses, interpretation, or writing of this manuscript.

## Results

The final analytical sample included 3840 individuals aged 51–100 years (Appendix p 4). Table 1 presents the 2016 characteristics for the whole cohort by BMI categories: BMI <25.0 kg/m^2^ (including 63 participants with a BMI < 18.5 kg/m^2^), BMI 25.0–29.9 kg/m^2^, and BMI ≥ 30.0 kg/m^2^. Characteristics by vital status at the end of the follow-up in December 2020 are shown in the Appendix (p 8). The distribution of baseline age and the distribution of BMI, as well as survival rates by age groups and survival rates by age, are presented in the Appendix (p 10–11).

**Table 1:** Characteristics of the analytical sample (n = 3840), and by BMI category.

Characteristics	Overall	BMI categories		
		<25.0 kg/m^2^	25.0–29.9 kg/m^2^	≥30.0 kg/m^2^
Sample size, n (%)	3840 (100)	1090 (28.4)	1412 (36.8)	1338 (34.8)
Follow-up time in years, mean (SD)	4.1 (0.8)	4.0 (0.9)	4.2 (0.7)	4.2 (0.7)
**Sociodemography**				
Baseline age in years, mean (SD)	69.9 (9.6)	72.6 (10.3)	70.4 (9.6)	67.3 (8.3)
Females, n (%)	2234 (58.2)	703 (64.5)	722 (51.1)	809 (60.5)
Education (High school grad and below), n (%)	1879 (48.9)	495 (45.4)	697 (49.4)	687 (51.3)
Smoking status in 2016, n (%)				
Never smokers	1687 (43.9)	460 (42.2)	634 (44.9)	593 (44.3)
Ever smokers	1710 (44.5)	473 (43.4)	616 (43.6)	621 (46.4)
Current smokers	443 (11.5)	157 (14.4)	162 (11.5)	124 (9.3)
Ethnicity/race, n (%)				
White/Caucasian	2891 (75.3)	892 (81.8)	1072 (75.9)	927 (69.3)
Black/African American	647 (16.8)	132 (12.1)	211 (14.9)	304 (22.7)
Other	302 (7.9)	66 (6.1)	129 (9.1)	107 (8.0)
Unhealthy alcohol use in 2016, n (%)	247 (6.4)	64 (5.9)	102 (7.2)	81 (6.1)
Age in 2014 in years, mean (SD)	67.8 (9.7)	70.3 (10.3)	68.3 (9.6)	65.1 (8.4)
Smoking status in 2014, n (%)				
Never smokers	1650 (43.8)	451 (41.8)	619 (44.9)	580 (44.2)
Ever smokers	1645 (43.6)	455 (42.2)	590 (42.8)	600 (45.7)
Current smokers	476 (12.6)	173 (16.0)	171 (12.4)	132 (10.1)
**Metabolic measures**				
BMI in kg/m^2^, mean (SD)	28.7 (6.1)	22.4 (2.1)	27.3 (1.4)	35.3 (5.0)
Hypertension, n (%)	2440 (63.6)	558 (51.2)	879 (62.3)	1003 (75.0)
Hyperglycemia, n (%)	1752 (45.6)	327 (30.0)	642 (45.5)	783 (58.5)
Hypertriglyceridemia, n (%)	1146 (29.9)	188 (17.3)	439 (31.1)	519 (38.8)
Low HDL, n (%)	1010 (26.3)	154 (14.1)	382 (27.1)	474 (35.4)
N of metabolic deficiencies, mean (SD)	1.7 (1.2)	1.1 (1.0)	1.7 (1.1)	2.1 (1.1)
Metabolically unhealthy, n (%)	3177 (82.7)	751 (68.9)	1191 (84.3)	1235 (92.3)
Metabolically unhealthy—less strict, N (%)	1973 (51.4)	344 (31.6)	727 (51.5)	902 (67.4)
BMI in 2014 in kg/m^2^, mean (SD)	28.9 (6.23)	23.0 (2.7)	27.5 (2.4)	35.1 (5.5)
Metabolically unhealthy in 2014, n (%)	3060 (81.1)	776 (71.9)	1115 (80.8)	1169 (89.1)
**Epigentic age acceleration**				
Horvath age acceleration-I, mean (SD)	0.0 (6.5)	−0.1 (6.5)	−0.2 (6.4)	0.3 (6.5)
Horvath age acceleration-II, mean (SD)	0.0 (4.4)	−0.3 (4.5)	−0.1 (4.5)	0.3 (4.3)
Hannum age acceleration, mean (SD)	0.0 (5.3)	−0.2 (5.5)	−0.2 (5.2)	0.3 (5.2)
PhenoAge acceleration, mean (SD)	0.0 (6.9)	−0.3 (7.2)	−0.4 (6.8)	0.7 (6.6)
GrimAge acceleration, mean (SD)	0.0 (4.8)	−0.2 (5.2)	−0.1 (4.9)	0.3 (4.3)
DunedinPACE, mean (SD)	1.1 (0.1)	1.1 (0.1)	1.1 (0.1)	1.1 (0.1)
**Comorbididities**				
History of cancer diagnosis, n (%)	643 (16.8)	198 (18.2)	244 (17.3)	201 (15.0)
History of lung disease diagnosis, n (%)	483 (12.6)	152 (13.9)	144 (10.2)	187 (14.0)
History of cardiovascular disease diagnosis, n (%)	1084 (28.3)	312 (28.6)	360 (25.5)	412 (30.9)
**Vital Status in 2020**				
Deceased, n (%)		176 (16.1)	133 (9.4)	114 (8.5)

Participants were categorised BMI < 25.0 kg/m^2^, 25.0–29.9 kg/m^2^, and ≥30.0 kg/m^2^.

BMI—body mass index, HDL—high-density lipoproteins cholesterol, kg/m^2^—kilograms per square metre, n—number, SD—standard deviation.

### Mediation models—linear regression

**Fig. 3:**
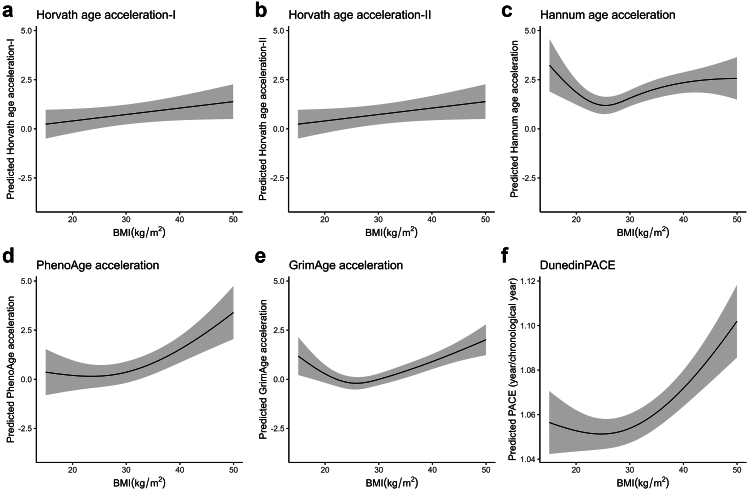
Association between BMI and epigenetic age acceleration from linear regression. Models were adjusted for age, sex, ethnicity/race, smoking status, educational attainment, and metabolic health (n = 3840). Shaded areas around the curves depict the confidence intervals. The plots were based on predictions made among white males aged 70 who were never smokers, metabolically unhealthy, and had a high school educational attainment or below. The association between BMI and epigenetic age acceleration for Horvath-I (panel a) was not significant. The associations between BMI and the other epigenetic age acceleration measures were significant (panels b–f). Abbreviations: BMI—body mass index, kg/m^2^—kilograms per square metre.

The associations between age and EAA were linear, except for HorvathAgeAcc-II, where age was modelled using restricted cubic splines (Appendix p 12). Estimates yielded from linear models with weights did not differ from those without. Therefore, we used an unweighted linear model as the main model (Appendix p 13–14). BMI was associated with each EAA, although the association with HorvathAgeAcc-I [Beta-coefficient(β) = 0.03, 95% CI = −0.003–0.07] was not statistically significant (Fig. 3, Appendix p 14). The association of BMI with HorvathAgeAcc-II was linear (β = 0.04, 95% CI = 0.01–0.06) but nonlinear for other EAAs, with low and high BMI associated with higher EAA. BMI levels corresponding to the lowest predicted EAA (nadir) for HannumAgeAcc, PhenoAgeAcc, GrimAgeAcc, and DunedinPACE were 25.4 kg/m^2^, 23.5 kg/m^2^, 25.8 kg/m^2^, and 24.7 kg/m^2^, respectively. BMI levels higher than the nadirs were consistently associated with higher EAA across all four clocks. For BMI levels lower than nadirs, EAA was higher for HannumAgeAcc and GrimAgeAcc, and comparable to the nadir for PhenoAgeAcc and DunedinPace.

### Outcome models—Gompertz proportional hazards regression model

Fig. 2a illustrates the association between BMI and predicted mean life expectancy. BMI had a nonlinear association with survival, with the lowest life expectancies occurring at the higher and lower extremes of BMI (Appendix p 15). The BMI associated with the highest mean life expectancy was approximately 27 kg/m^2^.

Gompertz proportional hazards models were conducted with and without weights, and the differences in the estimates between the models were minor (Appendix, p 16–17). Hence, we applied unweighted Gompertz proportional hazards models. Fig. 2b–g illustrate the predicted mean life expectancy as a function of EAA, calibrated for white males aged 70, with a BMI of 25 kg/m^2^, metabolically unhealthy status, no smoking history, and high school education or lower.

HorvathAgeAcc-I and HorvathAgeAcc-II showed concave associations with survival, with reduced mean life expectancies even at lower acceleration levels (Fig. 2b and c). HannumAgeAcc also displayed a somewhat concave association, though lower HannumAgeAcc was still associated with higher life expectancies compared to higher HannumAgeAcc (Fig. 2d). PhenoAgeAcc, GrimAgeAcc, and DunedinPACE displayed a consistent, monotonous decline in life expectancy with higher acceleration levels (Fig. 2e–g, Appendix p 17). On the hazard scale, one standard deviation increase in PhenoAgeAcc, GrimAgeAcc and DunedinPACE was associated with an increased risk of mortality by approximately 32% [Hazards ratio (HR) = 1.32, 95% CI = 1.23–1.51], 78% (HR = 1.78, 95% CI = 1.54–1.96), and 40% (HR = 1.40, 95% CI = 1.25–1.56), respectively.

HorvathAgeAcc-I was excluded from the mediation analyses because it was not associated with BMI in the mediation models, precluding its role as a potential mediator. HorvathAgeAcc-II was excluded from the primary mediation models due to its weak (small effect size) and paradoxical (low EAA associated with lower life expectancy) relationship with survival. The results were included in the supplement for completeness (Appendix p 17).

### Mediation analyses—high BMI as exposure

**Fig. 4:**
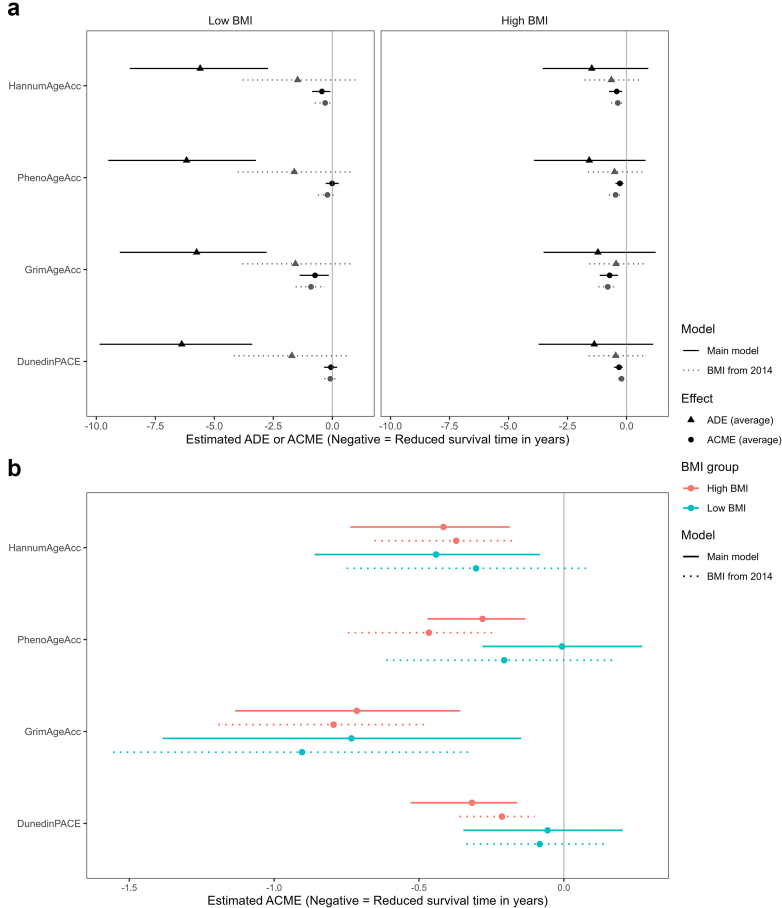
Mediation models of body mass index measured in 2016 (n = 3840) and 2014 (n = 3771), and survival time by epigenetic age acceleration. Average direct effects (ADE) and average causal mediation effects (ACME) were estimated from mediation analyses of the association between BMI and survival, with each epigenetic age acceleration as the mediator. Linear models were used to evaluate the association between BMI and respective mediator, and Gompertz proportional hazards models with age as timescale as outcome models. All models were adjusted for sex, ethnicity/race, smoking status, education, and metabolic health. Metabolically healthy status in BMI 2016 model was defined as the absence of hypertension, hyperglycemia, hypertriglyceridemia and low HDL. Metabolically healthy status in BMI 2014 model was defined as the absence of hypertension, hyperglycemia, and low HDL or self-reported high cholesterol levels. The x-axis shows the estimated mean difference in survival time. In Panel a, the left panel shows mean ADE (▲) and mean ACME (●) for low BMI defined with the exposed level at 19 kg/m^2^ and the unexposed level at 27 kg/m^2^, and the right panel shows the mean ADE and ACME for high BMI defined with the exposed level at 35 kg/m^2^ and the unexposed level at 27 kg/m. Black solid lines represent models using BMI self-reported in 2016. Grey dotted lines used the BMI self-reported in 2014. Panel b magnifies the ACMEs only for high (in blue) and low BMI (red). Solid lines represent models using BMI self-reported in 2016, and dotted lines represent models using BMI self-reported in 2014. Bars represent the 95% confidence intervals. Abbreviations: ACME—average causal mediation effects, ADE—average direct effects, BMI—body mass index.

Fig. 4 shows the ADE and ACME by each EAA in the association between BMI and survival. The confidence intervals for ADE appear wider than those of ACME, suggesting higher variation and potentially less precise estimates for ADEs. Fig. 4b zooms in on the ACME to facilitate the visualisation of the differences and confidence intervals of ACME by each EAA in the associations of low and high BMI with survival.

For the four remaining EAA, we found that the ADEs of high BMI (35 kg/m^2^ versus 27 kg/m^2^) were modest, reducing survival time by 1.21 to 1.58 years, but had wide CIs and were not statistically significant (Fig. 4, Appendix p 19). The mediated effects through the EAA ranged from a decrease of survival time by 0.28 to 0.71 years and were statistically significant for all EAAs. Specifically, HannumAgeAcc, PhenoAgeAcc, GrimAgeAcc, and DunedinPACE were estimated to mediate 22%, 15%, 37%, and 19% of the total high BMI-survival association, respectively.

### Mediation analyses—low BMI as exposure

For low BMI (19 kg/m^2^ versus 27 kg/m^2^), the ADEs on survival were large and statistically significant, reducing survival time by 5.60 to 6.38 years for all EAAs (Fig. 4, Appendix p 19). The mediated effects were smaller and statistically significant for HannumAgeAcc (ST reduction = 0.44, 95% CI = 0.08–0.86) and GrimAgeAcc (ST reduction = 0.73, 95% CI = 0.15–1.38), mediating 7% and 11% of the total effect of low BMI-survival effect, respectively.

### Sensitivity analyses

Results from using a less strict definition of metabolic health, adjusting further for a history of cancer, lung disease, or cardiovascular disease, and unhealthy alcohol use, and using self-reported BMI from 2014 were consistent directionally with the primary analyses, although there were slight attenuations in the magnitude of the effects (Appendix p 20–24).

Stratifying the mediation analyses by sex yielded findings that were consistent in direction in both males and females (Appendix p 26–27). When high BMI was the primary exposure, ADEs were stronger among men and ACMEs among women. When low BMI was the primary exposure, ADEs were substantially stronger among men.

## Discussion

In a nationally representative cohort of older adults from the US, high and low BMI were associated with accelerated epigenetic age and shorter survival time. The direct effects of low BMI were larger than those of high BMI, indicating a stronger reduction in survival time. The mediating role of EAA in the BMI-survival association varied by BMI levels. At high BMI, EAA, measured by four epigenetic clocks, mediated 15%–37% of the association with reduced survival time. In contrast, at low BMI, EAA from only two epigenetic clocks mediated the association, and to a lesser extent, at 7%–11%. To our knowledge, no previous study has evaluated the mediating role of epigenetic ageing in the BMI-mortality association, using EAA derived from different epigenetic clocks to capture the multidimensionality of biological ageing, thus enriching our understanding of the relationship between BMI, biological ageing, and survival.

Our findings are consistent with previous studies linking high BMI to increased EAA, although most did not evaluate the nonlinear relationship between BMI and EAA.^8^ We found that high and low BMI were associated with increased EAA, except for HorvathAgeAcc-I and HorvathAgeAcc-II. This nonlinear association mirrors the well-documented relationship between BMI and other health outcomes, with low and high BMI levels linked to worse health.^10^, ^11^, ^12^ Similarly, our study observed reduced survival at high and low BMI, consistent with prior research.^10^, ^11^, ^12^

Our findings support the hypothesis that obesity increases mortality risk by accelerating biological ageing. All four tested EAA measures mediated part of the high BMI-survival association, suggesting that obesity broadly accelerates cellular ageing. Since obesity and biological ageing share hallmarks, such as the accumulation of oxidative stress, chronic low-grade inflammation, mitochondrial dysfunction, and cellular senescence, it is plausible that obesity accelerates ageing processes through common biological pathways, thereby increasing the risk of age-related diseases and mortality.^1^^,^^2^ Further research should investigate these pathways, yielding knowledge crucial for developing targeted interventions that can mitigate or prevent the adverse effects of obesity on ageing and overall health.^1^^,^^2^

Like higher BMI, lower BMI was associated with higher EAA and shorter survival time. In contrast, low BMI had stronger direct effects on survival and less evidence for mediation through EAA than higher BMI. As such, the mechanisms linking high BMI to survival may differ from those linking low BMI to survival, and those that link low BMI to survival likely involve factors beyond biological ageing.

Since unintentional weight loss among older people is associated with increased mortality risk,^29^ the link between low BMI and mortality risk may largely be explained by unintended weight loss. Unintended weight loss may result from malabsorption, loss of lean body mass, and nutritional deficiencies triggered by underlying health conditions or medication use.^13^^,^^14^ Alternatively, it may result from age-related physiological changes, including reduced taste and smell, slower gastric emptying, quicker onset of satiety, and alterations in metabolism, leading to a faster rate of lean body mass loss.^13^^,^^14^ Therefore, as health deteriorates, whether due to age-related factors or comorbidities, unintentional weight loss may follow, leading to a lower BMI. Thus, low BMI may indicate health deterioration, explaining its stronger direct effects on survival. In this scenario, biological ageing likely plays a minor role in mediating the low BMI-survival relationship. On the other hand, biological ageing may contribute to processes leading to unintentional weight loss and, hence, lower BMI.

In contrast to high BMI, where significant mediation through all EAA was observed, the low BMI-survival association was significantly mediated only through HannumAgeAcc and GrimAgeAcc. It is plausible that GrimAgeAcc, an epigenetic clock trained to predict mortality.^8^ may better capture the processes linking low BMI and mortality than other EAAs that are not specifically trained for this purpose. That said, this interpretation does not explain the significant, albeit low, mediation effects through HannumAgeAcc, which was not specifically designed to predict mortality.

HannumAgeAcc, like HorvathAgeAcc-I and HorvathAgeAcc-II, are epigenetic clocks designed to predict chronological age rather than biological age.^30^ Hence, their associations with BMI and survival differed from those observed with other epigenetic clocks, which are tuned to predict biological ageing. For instance, the association between BMI and HorvathAgeAcc-I was not statistically significant, and paradoxically, lower values of HorvathAgeAcc-I and HorvathAgeAcc-II were associated with a higher mortality risk. The significant mediation effects of HannumAgeAcc in the low BMI-mortality association may thus reflect risks associated with chronological age instead of biological age.

Overall, there was weak evidence supporting EAA as a mediator between low BMI and survival. Nonetheless, the consistently stronger direct effects of EAA in the low BMI-survival link may reflect the role of low BMI from unintentional weight loss and health deterioration in reduced survival time. While these patterns of association and mediation between BMI, EAA and survival may be sex-specific, with stronger mediation through EAA in the high BMI-survival associations in females and stronger direct effects of both high and low BMI in males, the analyses were limited by low power from small sample size upon stratification. Future studies should further examine the role of sex in the inter-relationships between BMI, biological ageing, and survival.

The current study leveraged data from a nationally representative sample and a well-established cohort of older adults in the US to illuminate the role of EAA in the BMI-survival association. Additionally, we used several different measures of EAA, constructed to predict chronological age, age-related phenotypes, diseases, and mortality, to capture the complexity of biological ageing as good as possible. We carefully assessed nonlinearity in the associations, using spline terms where appropriate, and included BMI as a continuous variable to reduce loss of information from categorisation. This enabled us to evaluate the association between low BMI and high BMI and mortality, shedding light on two potentially distinct mechanisms that may affect survival. Furthermore, we incorporated objective measures from the Venous Blood Study and self-reported disease information to determine metabolic health status.

However, our study has limitations. Firstly, BMI was computed using self-reported height and weight, which may not accurately reflect actual measures. Nonetheless, previous studies have demonstrated that self-reported BMI can be a close proxy for measured BMI.^31^

Secondly, although we employed a prospective design by considering four-year mortality as the outcome, both exposures and mediators were measured cross-sectionally, which limited our ability to infer causality. Nevertheless, we conducted sensitivity analyses using BMI in 2014, which yielded findings consistent with the primary analyses. However, the analytical sample size using BMI in 2014 was smaller [n = 3771 with 412 deaths (10%)], and metabolic health measures used here differed from those used in the primary analyses.

The hypothesis that obesity accelerates biological ageing is substantiated by overlapping pathophysiological mechanisms between obesity and ageing and their associations with increased risk of similar age-related diseases, as evidenced from experimental research and observational studies.^1^^,^^2^ The majority of current research supports the notion that BMI changes most likely drive changes in DNA methylation patterns.^32^^,^^33^ Still, there remains uncertainty. A Mendelian randomisation study revealed a strong causal effect of obesity on epigenetic age, as well as evidence of a causal effect, albeit of smaller effect size, of epigenetic age on higher BMI.^34^ If EAA precedes and influences BMI, treating EAA as a mediator in the causal pathway could lead to biased estimates of ADE and ACME. Future research should explore the bi-directionality and temporal dynamics of this relationship.

Thirdly, this study's short follow-up time limits the ability to observe long-term effects. It increases the risk of biases where underlying illnesses may impact BMI, potentially leading to reverse causation. Fourthly, there may be selection bias, as individuals who completed a venous blood collection and were thus included in this study may differ systematically from those who did not, therefore potentially affecting the representativeness of the study population. Fifthly, we acknowledge the potential for selective survival, where participants with higher BMIs in late life may represent a healthier subset, which then attenuates the associations at higher BMI levels. To explore whether pre-existing illness may explain the lower life expectancy observed at low BMI, we conducted additional analyses adjusting for history of cancer, lung disease and cardiovascular disease. These adjustments reduced life expectancy estimates at both high and low BMI, suggesting that chronic diseases may contribute to the observed associations across the entire BMI range, and not just in low BMI (Appendix p 29).

Sixthly, our study did not adjust for potentially important confounders, such as diet and medication use, which may influence BMI, epigentic ageing and survival. Therefore, we cannot rule out the possibility of residual confounding. Lastly, although we used several epigenetic clocks to capture biological ageing, we did not examine specific biological pathways linking obesity with ageing and survival. Future cohort studies should examine individual biological processes to map the connections between obesity, ageing, and survival.

Our findings align with the hypothesis that obesity accelerates biological ageing, which increases mortality risk, highlighting the potential incorporation of epigenetic age measures into clinical risk assessments for older individuals with high BMI. Besides facilitating the identification of those at greater risk, these measures could also serve as markers for monitoring the therapeutic response to obesity management and intervention efforts.

### Conclusions

Our study identified a nonlinear relationship where low and high BMI levels were associated with increased EAA and reduced survival time. Epigenetic ageing may mediate the BMI-mortality association; however, the underlying mechanisms may differ for individuals with high and low BMI. Mediation through EAA was stronger and more consistent in high BMI, supporting the hypothesis that obesity increases mortality risk by accelerating ageing. In contrast, the stronger direct effects of low BMI on survival, with weaker evidence of mediation through EAA, suggest that factors beyond accelerated ageing, such as unintentional weight loss due to pre-existing conditions, may play a greater role in the low BMI-mortality association.

## Contributors

PL and IKK conceived the study. PL, IKK, and AP designed the study. IKK, AP, JJ, SH, DF and ADA advised on the study design. AP and IKK advised on the statistical analysis plan and interpretation of the results. AP modified existing mediation software for statistical analysis. PL performed statistical analyses and drafted the first version of the manuscript. IKK and AP advised on the initial drafting of the manuscript. All authors have access to the data used in this project. PL, IKK, and AP verified the underlying data. All authors reviewed and interpreted the data. All authors contributed to the editing and writing of the manuscript. All authors reviewed and approved the final manuscript for submission.

## Data sharing statement

The Health and Retirement Study is a publicly available dataset accessible through the HRS website: https://hrs.isr.umich.edu/. Additional registration is required for access to sensitive data. Codes used in the analyses of this dataset is available upon request from the corresponding authors.

## Declaration of interests

IKK received research funding from the National Institute on Aging/National Institutes of Health, under grant numbers AG060470, AG089666 and AG081248. All other authors declare no conflict of interest.
