# Supplementary material for: The mediating role of epigenetic ageing in the nonlinear association between body mass index and survival: a prospective cohort analysis of the US Health and Retirement Study
Source: eBioMedicine. 2025 Aug 16;119:105883. doi: 10.1016/j.ebiom.2025.105883 (PMC12391497; doi:10.1016/j.ebiom.2025.105883)
Supplement: Supplementary Figs. S1–S8 and Tables S1–S18 [file mmc1.pdf]

Supplementary materials:

List of supplemental materials in order of appearance in the manuscript

**Supplementary Table 1. Criteria and thresholds used for individual metabolic health components**	p3
**Supplementary Figure 1. Flow chart for selection of the analytical sample**	p4
**Supplementary Table 2. Comparing missing and non-missing data**	p5
**Supplementary Figure 2. AIC of survival models with different distributions of time to event**	p6
**Supplementary Text 1. Equations for estimating average direct effects and average causally mediating effects**	p7
**Supplementary Table 3. Characteristics for the whole analytical sample and by vital status at the end of follow-up in December 2020**	p8
**Supplementary Figure 3. Distribution of baseline age (n = 3,840)**	p10
**Supplementary Figure 4. Distribution of BMI by age groups 50 – 59, 60 – 69, 70 – 79, 80 – 89, and 90 years and above**	p10
**Supplementary Figure 5. Distribution of 4-year survival rates by age groups 50 – 59, 60 – 69, 70 – 79, 80 – 89, and 90 years and above**	p11
**Supplementary Table 4. Results from likelihood ratio tests of nested models to test the linearity of age and BMI in mediation models - linear regression**	p12
**Supplementary Table 5. Weighted estimates of BMI and covariates in association with each epigenetic age acceleration measure from linear regression**	p13
**Supplementary Table 6. Estimates of BMI and covariates in association with each epigenetic age acceleration measure from multivariable linear regression**	p14
**Supplementary Table 7. Results from likelihood ratio tests of nested models to test the linearity of BMI and epigenetic ageing markers in outcome models**	p15
**Supplementary Table 8. Weighted association of BMI and each epigenetic age acceleration measure with survival**	p16
**Supplementary Table 9. Association of BMI and each epigenetic age acceleration measure with survival**	p17
**Supplementary Table 10. Average direct effects and average causally mediating effects of high and low BMI on survival time with each epigenetic age acceleration measure as the mediator**	p19
**Supplementary Table 11: Average direct effects and average causally mediating effect of high BMI on survival time with each epigenetic age acceleration measure as the mediator from sensitivity analyses by adjusting for metabolic health with less strict criteria, additionally for history of cancer, lung disease, or cardiovascular disease, and using BMI in 2014**	p20
**Supplementary Table 12: Average direct effects and average causally mediating effect of low BMI on survival time with each epigenetic age acceleration measure as the mediator from sensitivity analyses by adjusting for metabolic health with less strict criteria, additionally for history of cancer, lung disease, or cardiovascular disease, and using BMI in 2014**	p22

Supplementary Figure 6. Mediation models of body mass index and survival time by epigenetic age acceleration, further adjusted for strict, and less strict definitions of metabolic health, further adjusted for history of cancer, lung disease, or cardiovascular disease, and unhealthy alcohol use p24

Supplementary Table 13: Sex stratified, average direct effects and average causally mediating effect of high and low BMI on survival time with each epigenetic age acceleration measure as the mediator p26

Supplementary Figure 7. Mediation models of body mass index and survival time by epigenetic age acceleration in females and males. p27

Supplementary Figure 8. Association between body mass index with mean life expectancy in years adjusted for age, sex, ethnicity/race, smoking status, education and metabolic health, and further for history of cancer, lung disease, or cardiovascular disease p29

Supplementary Table 1. Criteria and thresholds used for individual metabolic health components

**Metabolic components**	**Diagnostic criteria**	
	Data collection wave 2016	Data collection wave 2014
**Hypertension**	Self-reported diagnosis of hypertension	Self-reported diagnosis of hypertension
**Hyperglycemia**	Fasting BG ≥ 100 mg/dL or	HbA1c ≥ 5.7
	Non-fasting BG ≥ 126 mg/dL	
	Self-reported type II diabetes	Self-reported type II diabetes
**Low HDL**	HDL-C < 40 mg/dl in males	HDL-C < 40 mg/dl in males
	HDL-C < 50 mg/dl in females	HDL-C < 50 mg/dl in females
		Self-reported high cholesterol
**Hypertriglyceridemia**	Fasting triglyceride levels ≥ 150 mg/dL or	No data
	Non-fasting triglyceride levels ≥ 186 mg/dL	

Criteria for ascertaining each component used to determine metabolic health status. Metabolic components were deemed present when any one of the diagnostic criteria was fulfilled. Metabolically healthy status was defined as the absence of any of the above four metabolic components, and metabolically unhealthy as the presence of at least one metabolic component. A less strict criterion, defined metabolically healthy status as the presence of no more than one metabolic health component, was applied as a sensitivity analysis. Metabolically healthy status in 2014 was ascertained based on the absence of hypertension, hyperglycemia or abnormal cholesterol levels based on HDL-C levels or self-reported high cholesterol.

Abbreviations: BG – venous blood glucose levels, HDL-C – high-density lipoproteins cholesterol, mg/dL – milligram per deciliter.

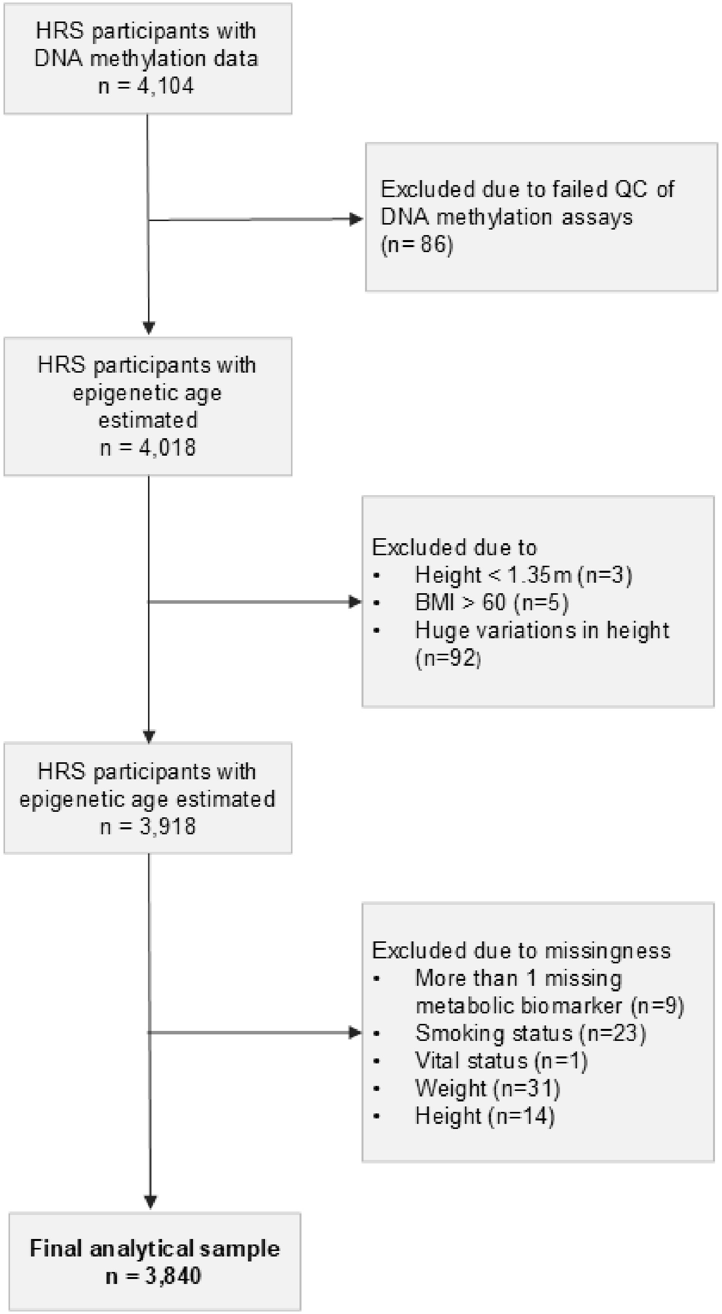

Supplementary Figure 1.

Flow chart for selection of the analytical sample.

Abbreviations: BMI – body mass index, DNA – deoxyribonucleic acid, HRS – Health Retirement Study, n – number, QC – quality control

Supplementary Table 2. Comparing missing and non-missing data

Characteristics	Complete cases	Missing	p-value
N (%)	3,931 (98·0)	78 (2·0)	
Follow-up time in years, mean (SD)	4·12 (0·75)	4·07 (0·75)	0·52
**Sociodemography**			
Baseline age in years, mean (SD)	69·45 (9·63)	69·62 (9·22)	0·88
Sex			
Females, N (%)	2,287 (58·2)	54 (69·2)	0·07
Males, N (%)	1,644 (41·8)	24 (30·8)	
Education			
High school grad and below, N (%)	1,949 (49·6)	41 (52·6)	0·68
College of above, N (%)	1,982 (50·4)	37 (47·4)	
Ethnicity/race, N (%)			
White/Caucasian	2,945 (74·9)	62 (79·5)	0·13
Black/African American	665 (16·9)	7 (9·0)	
Other	321 (8·2)	9 (11·5)	
Smoking status, N (%)			
Never smokers	1,731 (44·0)	27 (49·1)	0·59
Ever smokers	1,750 (44·5)	24 (43·6)	
Current smokers	450 (11·4)	4 (7·3)	
**Metabolic measures**			
BMI in kg/m^2, mean (SD)	28·69 (6·14)	28·94 (5·99)	0·78
Metabolic health, N (%)			
Metabolically healthy	674 (17·1)	7 (10·3)	0·18
Metabolically unhealthy	3,257 (82·9)	61 (89·7)	
**Epigentic age acceleration**			
Horvath age acceleration-I, mean (SD)	-0·02 (6·46)	1·05 (6·22)	0·15
Horvath age acceleration-II, mean (SD)	0·00 (4·44)	0·09 (4·10)	0·85
Hannum age acceleration, mean (SD	-0·02 (5·26)	0·91 (4·85)	0·12
PhenoAge acceleration, mean (SD)	-0·02 (6·85)	1·03 (6·01)	0·18
GrimAge acceleration, mean(SD)	0·00 (4·50)	-0·04 (3·87)	0·94
DunedinPACE, mean (SD)	1·07 (0·09)	1·08 (0·08)	0·37
Vital status, N (%)			
Alive	3,502 (89·1)	71 (91·0)	0·72
Deceased	429 (10·9)	7 (9·0)	

Continuous variables were compared with Student's t-test and categorical variables with the chi-square test. Fisher's exact test was performed for smoking status.

Abbreviations: BMI – body mass index, kg/m^2 – kilograms per square meter, N – number, SD – standard deviation

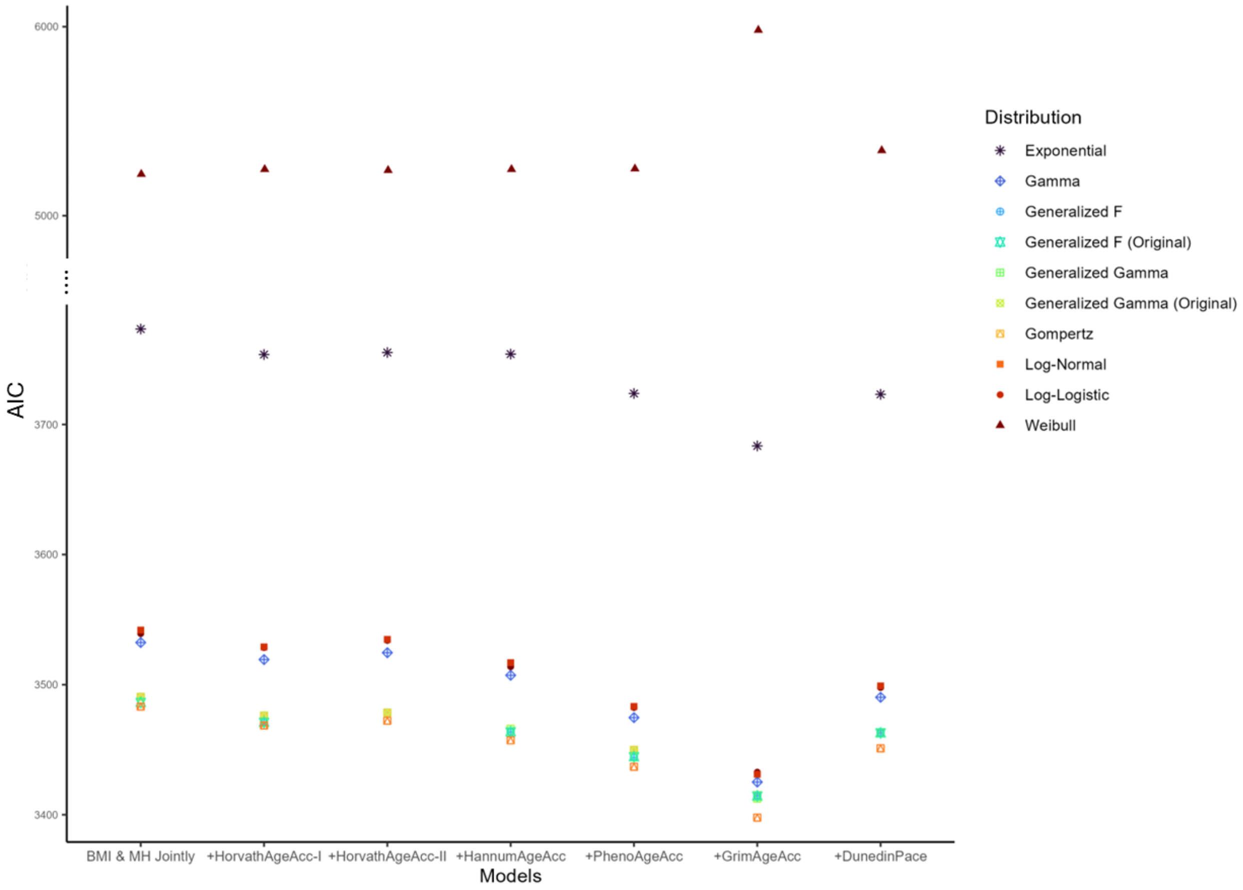

Supplementary Figure 2.

AIC of survival models with different distributions of time to event

Models included BMI and each epigenetic age acceleration, adjusted for age, sex, ethnicity/race, smoking status, educational attainment, and metabolic health status (n = 3,840). Y-axis shows the Akaike information criteria, not drawn to scale, due to the relatively larger AICs generated from models with Weibull distribution. The types of distribution used were exponential, gamma, generalised F, generalised F (original), generalised gamma, generalised gamma (original), Gompertz, log-normal, log-logistic, and Weibull, as listed in the legend.

Abbreviations: AIC - Akaike information criteria

Supplementary Text 1. Equations for estimating average direct effects and average causally mediating effects

Using high BMI as an example, we present how ADE and ACME were estimated by the corresponding equations:

$$ADE_{35\rightarrow 27} = \; Y_i\big(BMI\;(35),\; EAA_i(27)\big) - \; Y_i\big(BMI\;(27),\; EAA_i(27)\big)$$

where $ADE_{35\rightarrow 27}$ represents the direct effects of high BMI at 35kg/m^2 compared to the reference at 27kg/m^2. Y_i denotes the potential outcome of an individual. $Y_i\big(BMI\;(35),\; EAA_i(27)\big)$ represents the potential outcome (mean survival time) when BMI is at 35kg/m^2, and EAA is held constant at 27kg/m^2, whereas $Y_i\big(BMI\;(27),\; EAA_i(27)\big)$ is the potential outcome when BMI and EAA are at the reference levels. ADE is, therefore, the difference in potential outcomes when the mediator is held constant at a BMI of 27kg/m^2 and the BMI changes from 35kg/m^2 to 27kg/m^2.

ACME is expressed as follows:

$$ACME_{35\rightarrow 27} = Yi\big(BMI(27), EAAi(35)\big) - Yi\big(BMI(27), EAAi(27)\big)$$

where $ACME_{35\rightarrow 27}$ represents the ACME for high BMI at 35kg/m^2 compared to the reference at 27kg/m^2. $Y_i\big(BMI\;27, EAA_i(35)\big)$ denotes the potential outcome when BMI is fixed at 27kg/m^2 and EAA at a high BMI of 35kg/m^2. $Y_i\big(BMI\;27,\; EAA_i(27)\big)$ denotes the potential outcome when BMI is fixed at the reference level and EAA at the reference level. ACME is, therefore, the difference in potential outcomes when BMI is held constant at 27kg/m^2, and the BMI changes from 35kg/m^2 to 27kg/m^2.

Supplementary Table 3. Characteristics for the whole analytical sample and by vital status at the end of follow-up in December 2020

Characteristics	Overall	Alive	Deceased
N (%)	3,840 (100)	3,417 (89·0)	423 (11·0)
Follow-up time in years, mean (SD)	4·1	4·3	2·4
**Sociodemography**			
Baseline age in years, mean (SD)	69·9 (9·6)	68·9 (9·0)	78·2 (10·3)
Females, N (%)	2,234 (58·2)	2,011 (58·9)	223 (52·7)
Education (High school grad and below), N (%)	1,879 (48·9)	1,601 (46·9)	278 (65·7)
Smoking status in 2016, N (%)			
Never smokers	1,687 (43·9)	1,540 (45·1)	147 (34·8)
Ever smokers	1,710 (44·5)	1,494 (43·7)	216 (51·1)
Current smokers	443 (11·5)	383 (11·2)	60 (14·2)
Ethnicity/race, N (%)			
White/Caucasian	2,891 (75·3)	2,557 (74·8)	334 (79·0)
Black/African American	647 (16·8)	576 (16·9)	71 (16·8)
Other	302 (7·9)	284 (8·3)	18 (4·3)
Unhealthy alcohol use in 2016, N (%)			
Unhealthy alcohol use	247 (6·4)	231 (6·8)	16 (3·8)
No unhealthy alcohol use	3,584 (93·6)	3,600 (93·2)	3,815 (96·2)
Age in 2014 in years, mean (SD)	67·8 (9·7)	66·8 (9·1)	76·0 (10·2)
Smoking status in 2014, N (%)			
Never smokers	1,650 (43·8)	1,509 (44·9)	141 (34·2)
Ever smokers	1,645 (43·6)	1,441 (42·9)	204 (49·5)
Current smokers	476 (12·6)	409 (12·2)	67 (16·3)
**Metabolic measures**			
BMI in kg/m^2, mean (SD)	28·7 (6·1)	28·9 (6·1)	27·1 (6·2)
Hypertension, N (%)	2,440 (63·6)	2,109 (61·0)	331 (78·4)
Hyperglycemia, N (%)	1,752 (45·6)	1,526 (44·7)	226 (53·4)
Hypertriglyceridemia, N(%)	1,146 (29·9)	1,035 (30·3)	111 (26·3)
Low HDL, N (%)	1,010 (26·3)	872 (25·5)	138 (32·6)
N of metabolic deficiencies, mean (SD)	1·7 (1·2)	1·6 (1·2)	1·9 (1·2)
Metabolically unhealthy, N (%)	3,177 (82·7)	2,803 (82·0)	374 (88·4)
Metabolically unhealthy – less strict, N (%)	1,973 (51·4)	1,710 (50·0)	264 (62·2)
BMI in 2014 in kg/m^2, mean (SD)	28·9 (6·23)	29·0 (6·21)	27·8 (6·29)
Metabolically unhealthy in 2014, N (%)	3,060 (81·1)	2,693 (80·2)	367 (89·1)
**Epigentic age acceleration**			
Horvath age acceleration-I, mean (SD)	0·0 (6·5)	-0·1 (6·2)	0·6 (8·1)
Horvath age acceleration-II, mean (SD)	0·0 (4·4)	0·0 (4·3)	0·3 (5·5)
Hannum age acceleration, mean (SD	0·0 (5·3)	-0·2 (5·2)	1·3 (5·7)
PhenoAge acceleration, mean (SD)	0·0 (6·9)	-0·3 (6·7)	2·4 (7·6)
GrimAge acceleration, mean(SD)	0·0 (4·8)	-0·3 (4·7)	2·5 (4·9)
DunedinPACE, mean (SD)	1·1 (0·1)	1·1 (0·1)	1·1 (0·1)
**Comorbididities**			
History of cancer diagnosis, N (%)	643 (16·8)	516 (15·1)	127 (30·1)
History of lung disease diagnosis, N (%)	483 (12·6)	381 (11·2)	102 (24·2)
History of cardiovascular disease diagnosis, N (%)	1,084 (28·3)	881 (25·8)	203 (48·0)

Abbreviations: BMI – body mass index, HDL – high-density lipoproteins cholesterol, kg/m^2 – kilograms per square meter, N – number, SD – standard deviation

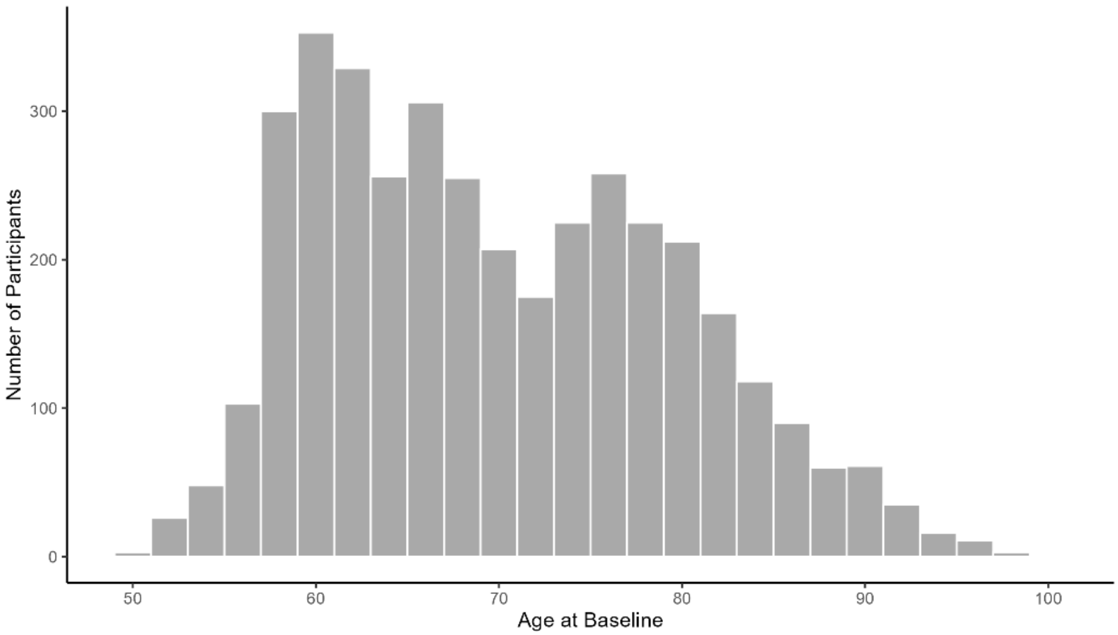

Supplementary Figure 3:

Distribution of baseline age (n = 3,840)

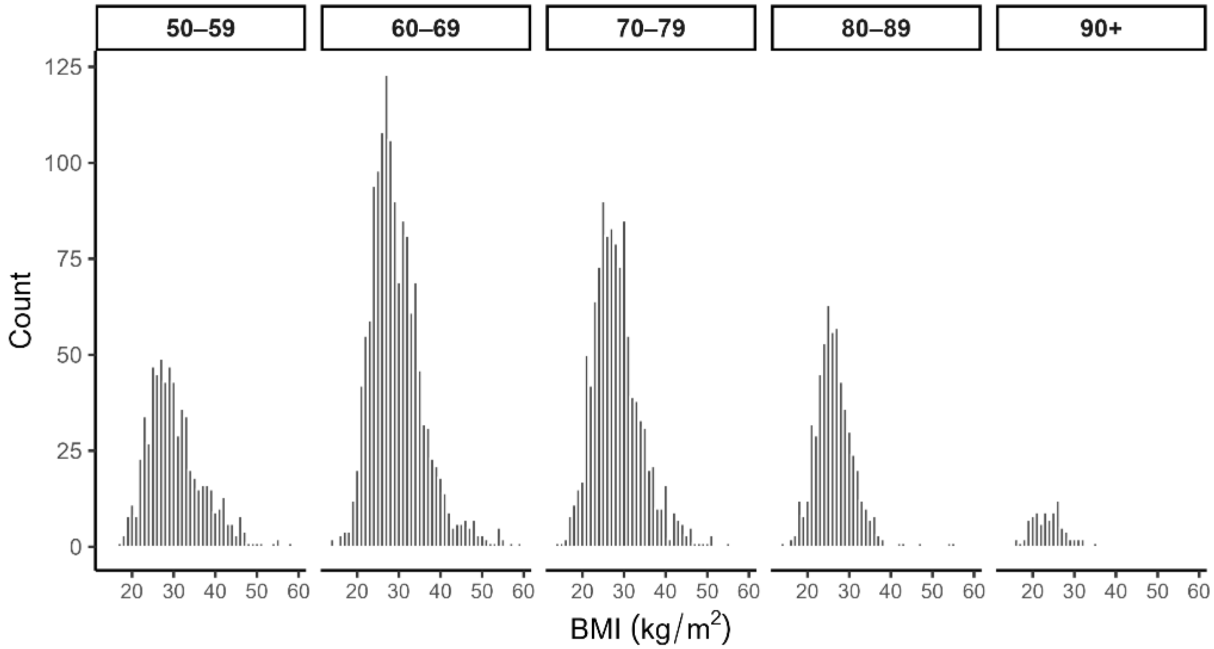

Supplementary Figure 4.

Distribution of BMI by age groups 50 – 59, 60 – 69, 70 – 79, 80 – 89, and 90 years and above (n = 3,840).

Abbreviation: BMI – body mass index, kg/m^2 – kilograms per square meter

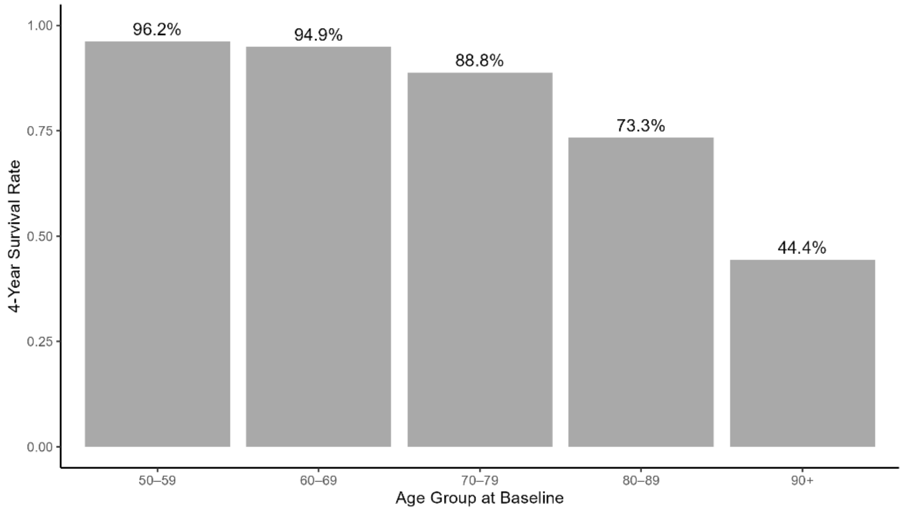

Supplementary Figure 5.

Distribution of 4-year survival rates by age groups 50 – 59, 60 – 69, 70 – 79, 80 – 89, and 90 years and above (n = 3,840)

Supplementary Table 4. Results from likelihood ratio tests of nested models to test the linearity of age and BMI in mediation models - linear regression

**Variables**		**Age**		**BMI**	
**Outcomes**	**Model**	**AIC**	**p-value LRT**	**AIC**	**p-value LRT**
**HorvathAgeAcc-I**	Linear	23,455	Base model	25,197	Base model
	2df	25,201	0·23	25,198	0·57
	3df	25,202		25,199	
	4df	25,203		25,199	
	5df	25,198		25,200	
**.HorvathAgeAcc-II**	Linear	22,284	Base model	22,240	Base model
	2df	22,250	2·67e-09	22,242	0·94
	3df	22,252		22,243	
	4df	22,254		22,244	
	5df	22,254		22,246	
**HannumAgeAcc**	Linear	23,455	Base model	23,443	Base model
	2df	23,456	0·39	23,442	0·10
	3df	23,456		23,434	0·001
	4df	23,458		23,436	
	5df	23,460		23,438	
**PhenoAgeAcc**	Linear	25,648	Base model	25,620	Base model
	2df	25,650	0·62	25,619	0·046
	3df	25,651		25,618	0·06
	4df	25,653		25,620	
	5df	25,653		25,622	
**GrimAgeAcc**	Linear	21,056	Base model	21,011	Base model
	2df	21,058	0·60	21,002	5·80e-4
	3df	21,056	0·11	20,999	2·36e-4
	4df	21,058		21,001	
	5df	21,060		21,002	
**DunedinPACE**	Linear	-8,255	Base model	-8,297	Base model
	2df	-8,256	0·09	-8,303	3·40e-3
	3df	-8,256		-8,302	
	4df	-8,256		-8,301	
	5df	-8,256		-8,300	

The linearity of age and BMI were tested by specifying them as restricted cubic splines (RCS) within linear regression models, with each EAA as the outcome (n = 3,840). All models were adjusted for age, sex, ethnicity/race, smoking status, educational attainment, and metabolic health. Presence of non-linearity was assessed by examining the AIC and performing likelihood ratio tests of nested models (LRT). We tested RCS with 2 to 5 degrees of freedom. The models with the lowest AIC were compared with the linear models, where the variable was specified in a linear form as the nested model. We also compared a simpler model with fewer degrees of freedom than the one with the lowest AIC with the linear model. A p-value of $< 0·05$ in the LRT indicates the models with RCS as significantly different and, therefore, suggests the presence of non-linearity.

Abbreviations: AIC - Aikake information criteria, BMI – body mass index, LRT – likelihood ratio tests of nested models, HorvathAgeAcc-I – acceleration of age predicted by Horvath-I epigenetic clocks, HorvathAgeAcc-II – acceleration of age predicted by Horvath-II epigenetic clocks, HannumAgeAcc – acceleration of age predicted by Hannum epigenetic clocks, PhenoAgeAcc – acceleration of age predicted by PhenoAge epigenetic clocks, GrimAgeAcc – acceleration of age predicted by GrimAge epigenetic clocks, DunedinPACE – rate of ageing in years per chronological year

Supplementary Table 5. Weighted estimates of BMI and covariates in association with each epigenetic age acceleration measure from linear regression

Outcomes	HorvathAgeAcc-I	HorvathAgeAcc-II	HannumAgeAcc	PhenoAgeAcc	GrimAgeAcc	DunedinPACE
**Variables**	**β (95% CI)**	**β (95% CI)**	**β (95% CI)**	**β (95% CI)**	**β (95% CI)**	**β (95% CI)**
**BMI or BMI spline 1**	0·05 (0·01,0·09)	0·04 (0·01,0·06)	0·74 (-0·23,1·71)	1·54 (-0·81,3·89)	0·50 (-0·23,1·23)	0·05 (0·02,0·08)
**BMI spline 2**			-0·65 (-3·88,2·58)	6·07 (3·77,8·37)	-0·90 (-3·32,1·53)	0·10 (0·08,0·13)
**BMI spline 3**			2·05 (-0·18,4·27)		3·06 (1·4,4·73)	
**MU (Reference: MH)**	0·29 (-0·23,0·82)	0·27 (-0·1,0·64)	0·33 (-0·09,0·75)	1·00 (0·42,1·57)	0·96 (0·64,1·27)	0·02 (0·01,0·02)
**Age or Age spline 1**	0·01 (-0·02,0·03)	2·20 (0·91,3·49)	0·001 (-0·02,0·02)	0·01 (-0·01,0·04)	0·05 (0·04,0·06)	0·001 (0·001,0·002)
**Age spline 2**		-1·69 (-2·64,-0·73)				
**Female (Reference: Males)**	-0·87 (-1·27,-0·46)	-0·78 (-1·07,-0·49)	-1·85 (-2·18,-1·52)	-1·17 (-1·62,-0·72)	-3·03 (-3·27,-2·78)	-0·01 (-0·02,-0·01)
**High school graduate and below (Reference: Above high school education)**	-0·03 (-0·46,0·39)	0·18 (-0·12,0·47)	0·29 (-0·05,0·62)	0·33 (-0·13,0·79)	0·86 (0·61,1·11)	0·01 (0,0·02)
**Currently smoking (Reference: Never smoked)**	0·30 (-0·4,1)	0·37 (-0·12,0·86)	0·97 (0·42,1·52)	1·33 (0·58,2·09)	7·62 (7·21,8·03)	0·12 (0·11,0·13)
**Ever smoked (Reference: Never smoked)**	0·19 (-0·24,0·62)	0·13 (-0·17,0·44)	0·01 (-0·33,0·35)	0·32 (-0·15,0·79)	1·93 (1·68,2·19)	0·02 (0·02,0·03)
**Race/ethnicity Black (Reference: White)**	0·005 (-0·67,0·68)	-0·39 (-0·86,0·08)	-1·98 (-2·51,-1·45)	0·62 (-0·11,1·35)	1·38 (0·98,1·78)	0·03 (0·02,0·03)
**Race/ethnicity Others (Reference: White)**	-0·14 (-1·00,0·72)	-0·07 (-0·67,0·53)	0·03 (-0·65,0·71)	0·32 (-0·62,1·25)	0·20(-0·31,0·71)	0·02 (0,0·03)

Weighted association between BMI and each epigenetic age acceleration measure from linear regression models adjusted for age, sex, educational attainment, smoking status, ethnicity/race, and metabolic health (n = 3,702).

Abbreviations: BMI – body mass index, CI – confidence intervals, β – beta-coefficient, MH – metabolically healthy, MU – metabolically unhealthy, HorvathAgeAcc-I – acceleration of age predicted by Horvath-I epigenetic clocks, HorvathAgeAcc-II – acceleration of age predicted by Horvath-II epigenetic clocks, HannumAgeAcc – acceleration of age predicted by Hannum epigenetic clocks, PhenoAgeAcc – acceleration of age predicted by PhenoAge epigenetic clocks, GrimAgeAcc – acceleration of age predicted by GrimAge epigenetic clocks, DunedinPACE – rate of ageing in years per chronological year

Supplementary Table 6. Estimates of BMI and covariates in association with each epigenetic age acceleration measure from linear regression

Outcomes	HorvathAgeAcc-I	HorvathAgeAcc-II	HannumAgeAcc	PhenoAgeAcc	GrimAgeAcc	DunedinPACE
**Variables**	**β (95% CI)**	**β (95% CI)**	**β (95% CI)**	**β (95% CI)**	**β (95% CI)**	**β (95% CI)**
**BMI or BMI spline 1**	0·03 (-0·003,0·07)	0·04 (0·01,0·06)	0·29 (-0·69,1·28)	2·15 (-0·16,4·47)	-0·07 (-0·79,0·64)	0·03 (4·29e-04,0·06)
**BMI spline 2**			-3·68 (-6·98,-0·37)	5·36 (3·11,7·62)	-1·22 (-3·61,1·17)	0·08 (0·06,0·11)
**BMI spline 3**			0·28 (-1·97,2·53)		2·62 (0·99,4·24)	
**MU (Reference: MH)**	0·53 (-0·04,1·09)	0·50 (0·11,0·88)	0·66 (0·21,1·12)	1·28 (0·68,1·88)	1·30 (0·97,1·62)	0·02 (0·01,0·02)
**Age or Age spline 1**	0·001 (-0·02,0·02)	2·45 (1·28,3·61)	-0·01 (-0·03,0·01)	0·01 (-0·01,0·04)	0·04 (0·03,0·05)	0·001 (7·02e-04,0·001)
**Age spline 2**		-2·08 (-3·06,-1·10)				
**Female (Reference: Males)**	-1·24 (-1·66,-0·82)	0·09 (-0·19,0·38)	-1·92 (-2·26,-1·58)	-0·87 (-1·31,-0·42)	-2·83 (-3·07,-2·58)	-0·01 (-0·02,-0·01)
**High school graduate and below (Reference: Above high school education)**	-0·01 (-0·43,0·41)	-1·07 (-1·35,-0·78)	0·21 (-0·12,0·54)	0·36 (-0·08,0·80)	0·66 (0·42,0·91)	0·01 (0·003,0·01)
**Currently smoking (Reference: Never smoked)**	0·37 (-0·32,1·07)	0·06 (-0·24,0·36)	0·75 (0·20,1·31)	1·64 (0·90,2·38)	7·80(7·40,8·20)	0·13 (0·12,0·14)
**Ever smoked (Reference: Never smoked)**	0·20 (-0·24,0·64)	0·49 (0·01,0·96)	0·01 (-0·34,0·36)	0·48 (0·02,0·95)	2·17 (1·92,2·42)	0·03 (0·02,0·04)
**Race/ethnicity Black (Reference: White)**	0·03 (-0·53,0·59)	-0·43 (-0·81,-0·05)	-2·17 (-2·62,-1·72)	0·04 (-0·55,0·64)	1·30 (0·98,1·62)	0·03 (0·02,0·03)
**Race/ethnicity Others (Reference: White)**	-0·26 (-1·04,0·53)	0·04 (-0·49,0·58)	0·21 (-0·41,0·83)	0·38 (-0·45,1·20)	0·10 (-0·35,0·55)	0·01 (0·002,0·02)

Association between BMI and each epigenetic age acceleration measure from linear regression models adjusted for age, sex, educational attainment, smoking status, ethnicity/race, and metabolic health (n = 3,840).

Abbreviations: BMI – body mass index, CI – confidence intervals, β – beta-coefficient, MH – metabolically healthy, MU – metabolically unhealthy, HorvathAgeAcc-I – acceleration of age predicted by Horvath-I epigenetic clocks, HorvathAgeAcc-II – acceleration of age predicted by Horvath-II epigenetic clocks, HannumAgeAcc – acceleration of age predicted by Hannum epigenetic clocks, PhenoAgeAcc – acceleration of age predicted by PhenoAge epigenetic clocks, GrimAgeAcc – acceleration of age predicted by GrimAge epigenetic clocks, DunedinPACE – rate of ageing in years per chronological year

Supplementary Table 7. Results from likelihood ratio tests of nested models to test the linearity of BMI and epigenetic ageing markers in outcome models – parametric survival models

Variables	Model	AIC	p-value LRT
**BMI**	Linear	3,496	Base
	2df	3,486	6·33-e04
	3df	3,484	2·52e-04
	4df	3,484	
	5df	3,485	
**HorvathAgeAcc-I**	Linear	3,482	Base
	2df	3,474	1·56e-03
	3df	3,469	1·56e-04
	4df	3,470	
	5df	3,471	
**HorvathAgeAcc-II**	Linear	3,482	Base
	2df	3,472	5·60e-4
	3df	3,474	
	4df	3,476	
	5df	3,478	
**HannumAgeAcc**	Linear	3,460	Base
	2df	3,460	0·13
	3df	3,457	0·03
	4df	3,459	
	5df	3,460	
**PhenoAgeAcc**	Linear	3,439	Base
	2df	3,441	0·76
	3df	3,437	0·054
	4df	3,438	
	5df	3,440	
**GrimAgeAcc**	Linear	3,399	Base
	2df	3,399	0·09
	3df	3,400	0·21
	4df	3,402	
	5df	3,403	
**DunedinPace**	Linear	3,451	Base
	2df	3,453	0·94
	3df	3,454	
	4df	3,456	
	5df	3,458	

The linearity of BMI and each EAA were tested by specifying them as restricted cubic splines (RCS) within Gompertz proportional hazards models with age as the underlying timescale (n = 3,840). All models were adjusted for sex, ethnicity/race, smoking status, educational attainment, and metabolic health. Models with each EAA were further adjusted for BMI. Presence of non-linearity was assessed by examining the AIC and performing likelihood ratio tests of nested models (LRT). We tested 2 to 5 degrees of freedom. The models with the lowest AIC were then compared to the nested linear model. A p-value of < 0·05 in the LRT indicates that the model with RCS is significantly different, suggesting the presence of non-linearity.

Abbreviations: AIC – Akaike information criteria, BMI – body mass index, df – degrees of freedom, LRT- likelihood tests, HorvathAgeAcc-I – acceleration of age predicted by Horvath-I epigenetic clocks, HorvathAgeAcc-II – acceleration of age predicted by Horvath-II epigenetic clocks, HannumAgeAcc – acceleration of age predicted by Hannum epigenetic clocks, PhenoAgeAcc – acceleration of age predicted by PhenoAge epigenetic clocks, GrimAgeAcc – acceleration of age predicted by GrimAge epigenetic clocks, DunedinPACE – rate of ageing in years per chronological year

Supplementary Table 8. Weighted association of BMI and each epigenetic age acceleration measure with survival

EAA included in the model	None	HorvathAgeAcc-I	HorvathAgeAcc-II	HannumAgeAcc	PhenoAgeAcc	GrimAgeAcc	DunedinPACE
**Variables**	**HR (95% CI)**	**HR (95% CI)**	**HR (95% CI)**	**HR (95% CI)**	**HR (95% CI)**	**HR (95% CI)**	**HR (95% CI)**
**BMI spline 1**	0·37 (0·37,1·25)	0·69 (0·38,1·28)	0·68 (0·37,1·26)	0·70 (0·38,1·29)	0·71 (0·38,1·31)	0·66 (0·36,1·21)	0·62 (0·34,1·14)
**BMI spline 2**	0·02 (0·02,0·46)	0·12 (0·03,0·53)	0·09 (0·02,0·42)	0·10 (0·02,0·46)	0·08 (0·02,0·38)	0·11 (0·03,0·5)	0·09 (0·02,0·39)
**BMI spline 3**	0·16 (0·16,3·34)	0·74 (0·16,3·45)	0·68 (0·15,3·17)	0·67 (0·14,3·17)	0·56 (0·12,2·69)	0·62 (0·13,2·88)	0·6 (0·13,2·77)
**EAA spline 1 or EAA**		0·77 (0·38,1·54)	0·33 (0·06,1·68)	4·88 (1·25,19·11)	1·04 (1·03,1·05)	1·13 (1·11,1·16)	1·65 (1·33,2·05)
**EAA spline 2**		0·06 (0·00,0·93)	2·32 (0·92,5·88)	2·00 (0·01,616·9)			48·08 (14·69,157·33)
**EAA spline 3**		1·39 (0·14,14·23)		1·29 (0·04,44·3)			0·85 (0·68,1·05)
**MU (Reference: MH)**	0·95 (0·95,1·75)	1·26 (0·93,1·7)	1·28 (0·95,1·74)	1·28 (0·95,1·74)	1·24 (0·92,1·68)	1·15 (0·85,1·56)	1·27 (0·94,1·72)
**Female (Reference: Male)**	0·65 (0·65,1·00)	0·80 (0·65,1)	0·81 (0·65,1)	0·87 (0·7,1·08)	0·8 (0·65,1)	1·19 (0·95,1·5)	1·5 (1·03,2·19)
**High school graduate and below (Reference: Above high school education)**	1·35 (1·35,2·08)	1·69 (1·36,2·1)	1·69 (1·36,2·09)	1·67 (1·35,2·07)	1·71 (1·38,2·12)	1·59 (1·29,1·98)	1·14 (0·91,1·43)
**Currently smoking (Reference: Never smoked)**	1·67 (1·67,3·35)	2·38 (1·68,3·38)	2·34 (1·65,3·33)	2·34 (1·65,3·32)	2·27 (1·6,3·22)	0·91 (0·61,1·35)	1·26 (0·92,1·72)
**Ever smoked (Reference: Never smoked)**	1·02 (1·02,1·59)	1·27 (1·02,1·59)	1·28 (1·03,1·6)	1·29 (1·03,1·61)	1·26 (1·01,1·58)	0·93 (0·74,1·18)	0·88 (0·5,1·56)
**Race/ethnicity Black (Reference: White)**	1·00 (1,1·85)	1·35 (0·99,1·84)	1·34 (0·99,1·83)	1·49 (1·09,2·04)	1·25 (0·91,1·7)	1·08 (0·79,1·48)	0·62 (0·34,1·14)
**Race/ethnicity Others (Reference: White)**	0·53 (0·53,1·66)	0·96 (0·54,1·69)	0·92 (0·52,1·62)	0·95 (0·54,1·68)	0·91 (0·52,1·6)	0·96 (0·54,1·7)	0·09 (0·02,0·39)

Hazards ratios were estimated from the Gompertz proportional hazards models that were weighted, with chronological age as the underlying time scale (n = 3,840). The models were adjusted for sex, ethnicity/race, smoking status, educational attainment, and metabolic health.

Abbreviations: BMI – body mass index, MH – metabolically healthy, MU – metabolically unhealthy, CI – confidence intervals, HR – hazard ratios, HorvathAgeAcc-I – acceleration of age predicted by Horvath-I epigenetic clocks, HorvathAgeAcc-II – acceleration of age predicted by Horvath-II epigenetic clocks, HannumAgeAcc – acceleration of age predicted by Hannum epigenetic clocks, PhenoAgeAcc – acceleration of age predicted by PhenoAge epigenetic clocks, GrimAgeAcc – acceleration of age predicted by GrimAge epigenetic clocks, DunedinPACE – rate of ageing in years per chronological year

Supplementary Table 9. Association of BMI and each epigenetic age acceleration measure with survival

EAA included in the model	None	HorvathAgeAcc-I	HorvathAgeAcc-II	HannumAgeAcc	PhenoAgeAcc	GrimAgeAcc	DunedinPACE
**Variables**	**HR (95% CI)**	**HR (95% CI)**	**HR (95% CI)**	**HR (95% CI)**	**HR (95% CI)**	**HR (95% CI)**	**HR (95% CI)**
**BMI spline 1**	0·58 (0·33,1·02)	0·59 (0·33,1·03)	0·57 (0·33,1·01)	0·6 (0·34,1·05)	0·58 (0·33,1·02)	-0·55(-1·11,0·003)	0·55 (0·31,0·96)
**BMI spline 2**	0·11 (0·02,0·45)	0·12 (0·03,0·52)	0·10 (0·02,0·42)	0·12 (0·03,0·5)	0·09 (0·02,0·4)	-2·16(-3·61,-0·72)	0·10 (0·02,0·41)
**BMI spline 3**	1·06 (0·28,3·95)	1·13 (0·3,4·29)	1·02 (0·27,3·86)	1·04 (0·27,4·00)	0·90 (0·23,3·48)	-0·08(-1·42,1·26)	0·92 (0·25,3·43)
**EAA spline 1 or EAA**		0·83 (0·44,1·55)	0·28 (0·06,1·24)	4·51 (1·51,13·47)	1·05 (1·03,1·06)	0·12(0·09,0·14)	27·97 (9·24,84·66)
**EAA spline 2**		0·04 (0·005,0·41)	5·19 (2·25,11·96)	0·40(0·004,36·97)			
**EAA spline 3**		1·52 (0·21,10·84)		0·61 (0·02,15·98)			
**MU (Reference: MH)**	1·36 (1·00,1·85)	1·31 (0·96,1·78)	1·33 (0·98,1·81)	1·34 (0·99,1·83)	1·31 (0·96,1·78)	0·18(-0·13,0·49)	1·33 (0·98,1·81)
**Female (Reference: Male)**	0·74 (0·61,0·91)	0·75 (0·62,0·92)	0·76 (0·62,0·93)	0·82 (0·67,1·01)	0·75 (0·62,0·92)	0·07(-0·15,0·28)	0·77 (0·63,0·95)
**High school graduate and below (Reference: Above high school education)**	1·57 (1·28,1·93)	1·59 (1·29,1·95)	1·57 (1·28,1·93)	1·56 (1·27,1·92)	1·57 (1·28,1·93)	0·41(0·21,0·62)	1·56 (1·27,1·91)
**Currently smoking (Reference: Never smoked)**	2·65 (1·91,3·67)	2·65 (1·91,3·67)	2·6 (1·88,3·6)	2·62 (1·89,3·62)	2·48 (1·80,3·44)	0·04(-0·34,0·42)	1·75 (1·23,2·50)
**Ever smoked (Reference: Never smoked)**	1·36 (1·10,1·69)	1·36 (1·09,1·68)	1·38 (1·12,1·71)	1·38 (1·11,1·71)	1·33 (1·08,1·65)	0·01(-0·22,0·24)	1·23 (0·99,1·53)
**Race/ethnicity Black (Reference: White)**	1·36 (1·05,1·77)	1·38 (1·06,1·8)	1·38 (1·06,1·79)	1·56 (1·2,2·05)	1·35 (1·04,1·75)	0·14(-0·12,0·41)	1·27 (0·98,1·66)
**Race/ethnicity Others (Reference: White)**	1·00 (0·62,1·62)	1·02 (0·63,1·65)	0·997 (0·62,1·61)	0·99 (0·61,1·61)	0·97 (0·6,1·57)	0·02(-0·46,0·50)	0·95 (0·59,1·54)

Hazards ratios were estimated from the Gompertz proportional hazards models with chronological age as the underlying time scale (n = 3,840). The models were adjusted for sex, ethnicity/race, smoking status, educational attainment, and metabolic health (n = 3,840).

Abbreviations: BMI – body mass index, MH – metabolically healthy, MU – metabolically unhealthy, CI – confidence intervals, HR – hazard ratios, HorvathAgeAcc-I – acceleration of age predicted by Horvath-I epigenetic clocks, HorvathAgeAcc-II – acceleration of age predicted by Horvath-II epigenetic clocks,

HannumAgeAcc – acceleration of age predicted by Hannum epigenetic clocks, PhenoAgeAcc – acceleration of age predicted by PhenoAge epigenetic clocks, GrimAgeAcc – acceleration of age predicted by GrimAge epigenetic clocks, DunedinPACE – rate of ageing in years per chronological year

Supplementary Table 10. Average direct effects and average causally mediating effects of high and low BMI on survival time with each epigenetic age acceleration measure as the mediator

		High BMI			Low BMI		
**Effects**	**Mediators**	**Est.**	**95% CI**	**Proportion mediated**	**Est.**	**95% CI**	**Proportion mediated**
**ADE**	HannumAgeAcc	-1·47	-3·55,0·92	0·22	-5·60	-8·58,-2·73	0·07
**ACME**		-0·42	-0·74,-0·19		-0·44	-0·86,-0·08	
**ADE**	PhenoAgeAcc	-1·58	-3·92,0·81	0·15	-6·17	-9·49,-3·24	0·001
**ACME**		-0·28	-0·47,-0·13		-0·01	-0·28,0·27	
**ADE**	GrimAgeAcc	-1·21	-3·52,1·23	0·37	-5·75	-9·00,-2·78	0·11
**ACME**		-0·71	-1·13,-0·36		-0·73	-1·38,-0·15	
**ADE**	DunedinPACE	-1·36	-3·73,1·13	0·19	-6·38	-9·86,-3·39	0·01
**ACME**		-0·32	-0·53,-0·16		-0·06	-0·35,0·20	
**ADE**	HorvathAgeAcc-II	-1·64	-3·91,0·73	0·04	-6·24	-9·44,-3·32	NA
**ACME**		-0·06	-0·15,-0·01		0·06	0·003,0·14	

Results from mediation analysis estimating direct effects of high and low BMI and mediating effects through epigenetic age acceleration measured from epigenetic clocks: HannumAge, PhenoAge GrimAge, DunedinPACE, and HorvathAgeAcc-II (n = 3,840). Estimate parameters are expressed as the mean difference in survival time, where a negative coefficient indicates a reduction in survival time. When *high* BMI was the main exposure, the average direct effects and average mediating effects represent the difference in survival time at the exposed level at a high BMI of 35kg/m^2 compared to the unexposed level set at a BMI of 27kg/m^2. When *low* BMI was the main exposure, the average direct effects and average mediating effects represent the difference in survival time at the exposed level at a low BMI of 19kg/m^2 compared to the unexposed level set at a BMI of 27kg/m^2. The associations between exposures and mediators were modeled by linear regression. The associations between exposure and mediators with time to mortality were modeled by parametric survival models with Gompertz distribution and chronological age as the underlying timescale. All models were adjusted for age, sex, educational attainment, smoking status, race/ethnicity, and metabolic health. Proportion mediated reported as NA if ADE and ACME did not align in the same direction.

Abbreviations: ACME – average causal mediating effects, ADE – average direct effects, BMI – body mass index, CI – confidence interval, Est. – estimate, HannumAgeAcc – acceleration of age predicted by Hannum epigenetic clocks, PhenoAgeAcc – acceleration of age predicted by PhenoAge epigenetic clocks, GrimAgeAcc – acceleration of age predicted by GrimAge epigenetic clocks, DunedinPACE – rate of ageing in years per chronological year, HorvathAgeAcc-II – acceleration of age predicted by HorvathAgeAcc epigenetic clocks

Supplementary Table 11: Average direct effects and average causally mediating effect of high BMI on survival time with each epigenetic age acceleration measure as the mediator from sensitivity analyses by adjusting for metabolic health with less strict criteria, additionally for history of cancer, lung disease, or cardiovascular disease, and using BMI in 2014

Types of Models		Adjusted for metabolic health with less strict criteria		Additionally adjusted for history of cancer		Additionally adjusted for history of lung disease		Additionally adjusted for history of cardiovascular disease		Additionally adjusted for unhealthy alcohol use		BMI in 2014	
**Sample size**		3,840		3,837		3,839		3,836		3,831		3,771	
**Mediators**	**Effects**	**Est.**	**95% CI**	**Est.**	**95% CI**	**Est.**	**95% CI**	**Est.**	**95% CI**	**Est.**	**95% CI**	**Est.**	**95% CI**
**HannumAgeAcc**	ADE	-0·88	-2·98,1·53	-1·61	-3·88,0·85	-1·20	-3·49,1·23	-1·28	-3·5,1·21	-1·51	-3·67,0·89	-0·76	-1·85,0·43
	ACME	-0·34	-0·64,-0·13	-0·40	-0·69,-0·18	-0·40	-0·70,-0·19	-0·40	-0·70,-0·18	-0·41	-0·73,-0·19	-0·37	-0·64,-0·16
	Prop. mediated	0·28		0·20		0·25		0·24		0·21		0·33	
**PhenoAgeAcc**	ADE	-0·97	-3·31,1·48	-1·73	-4·14,0·78	-1·30	-3·64,1·09	-1·43	-3·78,0·93	-1·64	-3·88,0·91	-0·61	-1·69,0·61
	ACME	-0·22	-0·39,-0·08	-0·28	-0·46,-0·13	-0·26	-0·44,-0·12	-0·25	-0·44,-0·11	-0·29	-0·48,-0·14	-0·46	-0·74,-0·24
	Prop. mediated	0·18		0·14		0·17		0·15		0·15		0·43	
**GrimAgeAcc**	ADE	-0·62	-2·93,1·86	-1·34	-3·77,1·19	-1·04	-3·41,1·38	-1·04	-3·42,1·51	-1·26	-3·5,1·16	-0·44	-1·59,0·87
	ACME	-0·57	-0·95,-0·24	-0·72	-1·14,-0·37	-0·60	-0·98,-0·27	-0·62	-1·02,-0·28	-0·73	-1·14,-0·38	-0·79	-1·19,-0·48
	Prop. mediated	0·48		0·35		0·36		0·37		0·36		0·64	
**DunedinPACE**	ADE	-0·70	-3·08,1·92	-0·31	-0·51,-0·16	-1·04	-3·38,1·61	-1·22	-3·74,1·44	-1·42	-3·84,1·04	-0·55	-1·71,0·70
	ACME	-0·26	-0·45,-0·12	-1·55	-3·89,0·96	-0·30	-0·49,-0·15	-0·29	-0·49,-0·14	-0·32	-0·53,-0·16	-0·24	-0·41,-0·12
	Prop. mediated	0·27		0·17		0·22		0·19		0·18		0·31	

Results from mediation analysis estimating direct effects of high BMI and mediating effects through epigenetic age acceleration measured from epigenetic clocks: HannumAge, PhenoAge GrimAge, and DunedinPACE. Estimated parameters are expressed as the difference in survival time. The average direct effects and average mediating effects represent the difference in survival time at the exposed level set at a high BMI of 35kg/m^2 compared to the unexposed level set at a BMI of 27kg/m^2. The associations between exposures and mediators were modelled by linear regression. The associations between exposure and mediators with time to mortality were modelled using parametric survival models with Gompertz distribution and chronological age as the underlying timescale. Proportion mediated was computed only when ADE and ACME; otherwise, it was reported as NA. All models were adjusted for sex, educational attainment, smoking status, race/ethnicity, and metabolic health. Metabolically healthy status was defined as the absence of hypertension, hyperglycemia, hypertriglyceridemia and low high-density lipoprotein cholesterol. Metabolic health with less strict criteria defined metabolically healthy status as: no more than one (≤1) of hypertension, hyperglycemia, hypertriglyceridemia, and low high-density lipoprotein cholesterol. Models additionally adjusted for history of diseases or unhealthy alcohol use were adjusted for metabolic health using the strict criteria. When using BMI in 2014, metabolically healthy was defined as the absence of hypertension, hyperglycemia, and low high-density lipoprotein cholesterol or self-reported high cholesterol levels.

Abbreviations: ACME – average causal mediating effects, ADE – average direct effects, BMI – body mass index, CI – confidence interval, Est. – estimate, Prop. Mediated – proportion mediated, HannumAgeAcc – acceleration of age predicted by Hannum epigenetic clocks, PhenoAgeAcc – acceleration of age predicted by PhenoAge epigenetic clocks, GrimAgeAcc – acceleration of age predicted by GrimAge epigenetic clocks, DunedinPACE – rate of ageing in years per chronological year

Supplementary Table 12: Average direct effects and average causally mediating effect of low BMI on survival time with each epigenetic age acceleration measure as the mediator from sensitivity analyses by adjusting for metabolic health with less strict criteria, additionally for history of cancer, lung disease, or cardiovascular disease, and using BMI in 2014

**Types of Models**		**Adjusted for metabolic health with less strict criteria**		**Additionally adjusted for history of cancer**		**Additionally adjusted for history of lung disease**		**Additionally adjusted for history of cardiovascular disease**		**Additionally adjusted for unhealthy alcohol use**		**BMI in 2014**	
**Sample size**		3,840		3,837		3,839		3,836		3,831		3,771	
**Mediators**	**Effects**	**Est.**	**95% CI**	**Est.**	**95% CI**	**Est.**	**95% CI**	**Est.**	**95% CI**	**Est.**	**95% CI**	**Est.**	**95% CI**
**HannumAgeAcc**	ADE	-6·72	-9·72,-3·77	-5·59	-8·86,-2·67	-5·32	-8·47,-2·38	-6·14	-9·33,-3·18	-5·58	-8·78,-2·81	-1·38	-3·64,0·96
	ACME	-0·47	-0·88,-0·13	-0·40	-0·85,-0·05	-0·42	-0·85,-0·07	-0·43	-0·86,-0·09	-0·43	-0·89,-0·08	-0·27	-0·69,0·12
	Prop. mediated	0·06		0·07		0·07		0·07		0·07		0·16	
**PhenoAgeAcc**	ADE	-7·31	-10·71,-4·24	-6·12	-9·62,-3·03	-5·98	-9·21,-3·07	-6·65	-9·93,-3·51	-6·16	-9·29,-3·31	-1·55	-3·95,0·90
	ACME	-0·08	-0·35,0·19	0·01	-0·27,0·30	0·02	-0·24,0·30	-0·02	-0·29,0·27	-0·005	-0·28,0·28	-0·19	-0·58,0·17
	Prop. mediated	0·01		NA		NA		0·003		0·001		0·11	
**GrimAgeAcc**	ADE	-6·85	-10·24,-3·87	-5·61	-9·13,-2·59	-5·52	-8·83,-2·59	-6·13	-9·43,-3·17	-5·76	-9·1,-2·92	-1·56	-3·81,0·77
	ACME	-0·81	-1·47,-0·24	-0·69	-1·35,-0·11	-0·56	-1·18,-0·03	-0·69	-1·36,-0·08	-0·73	-1·38,-0·13	-0·90	-1·56,-0·31
	Prop. mediated	0·11		0·11		0·09		0·10		0·11		0·37	
**DunedinPACE**	ADE	-7·49	-11·06,-4·39	-6·29	-9·88,-3·11	-6·08	-9·51,-2·98	-6·89	-10·51,-3·74	-6·38	-9·90, -3·30	-1·63	-3·96,0·72
	ACME	-0·11	-0·40,0·14	-0·04	-0·31,0·23	-0·03	-0·31,0·22	-0·07	-0·34,0·19	-0·04	-0·32,0·22	-0·03	-0·25,0·21
	Prop. mediated	0·01		0·01		0·01		0·01		0·01		0·02	

Results from mediation analysis estimating direct effects of low BMI and mediating effects through epigenetic age acceleration measured from epigenetic clocks: HannumAge, PhenoAge GrimAge, and DunedinPACE. Estimated parameters are expressed as the difference in survival time. The average direct effects and average mediating effects represent the difference in survival time at the exposed levels set at a low BMI of 19kg/m^2 compared to the unexposed level set at a BMI of 27kg/m^2. The associations between exposures and mediators were modelled by linear regression. The associations between exposure and mediators with time to mortality were modelled using parametric survival models with Gompertz distribution and chronological age as the underlying timescale. Proportion mediated was computed only when ADE and ACME; otherwise, it was reported as NA. All models were adjusted for sex, educational attainment, smoking status, race/ethnicity, and metabolic health. Metabolically healthy status was defined as the absence of hypertension, hyperglycemia, hypertriglyceridemia and low high-density lipoprotein cholesterol. Metabolic health with less strict criteria defined metabolically healthy status as: no more than one (≤1) of hypertension, hyperglycemia, hypertriglyceridemia, and low high-density lipoprotein cholesterol. Models additionally adjusted for history of diseases or unhealthy alcohol use were adjusted for metabolic health using the strict criteria. When using BMI in 2014, metabolically healthy status was defined as the absence of hypertension, hyperglycemia, and low high-density lipoprotein cholesterol or self-reported high cholesterol levels.

Abbreviations: ACME – average causal mediating effects, ADE – average direct effects, BMI – body mass index, CI – confidence interval, Est. – estimates, Prop. mediated – proportion mediated, HannumAgeAcc – acceleration of age predicted by Hannum epigenetic clocks, PhenoAgeAcc – acceleration of age predicted by

PhenoAge epigenetic clocks, GrimAgeAcc – acceleration of age predicted by GrimAge epigenetic clocks, DunedinPACE – rate of ageing in years per chronological year, NA – not applicable

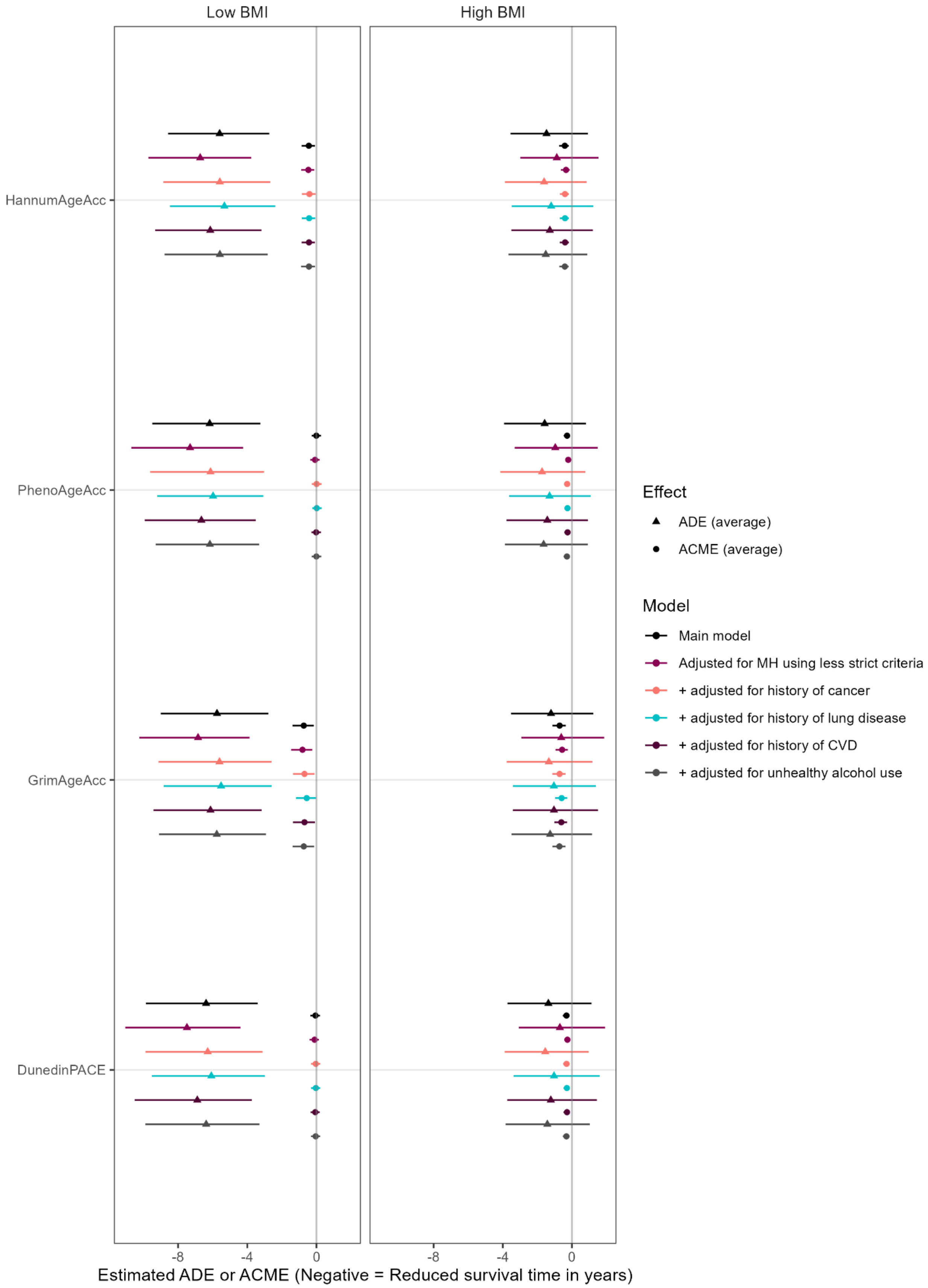
Low BMI
High BMI
HannumAgeAcc
PhenoAgeAcc
GrimAgeAcc
DunedinPACE
-8
-4
0
-8
-4
0
Estimated ADE or ACME (Negative = Reduced survival time in years)
Effect
ADE (average)
ACME (average)
Model
Main model
Adjusted for MH using less strict criteria
+ adjusted for history of cancer
+ adjusted for history of lung disease
+ adjusted for history of CVD
+ adjusted for unhealthy alcohol use

Supplementary Figure 6.

Mediation models of body mass index and survival time by epigenetic age acceleration, further adjusted for strict (n = 3,840) and less strict (n = 3,840) definitions of metabolic health, further adjusted for history of cancer (n = 3,837), lung disease (n = 3,839), or cardiovascular disease (n = 3,836), and unhealthy alcohol use (n = 3,831).

Average direct effects (ADE) and average causally mediating effects (ACME) were estimated from mediation analyses of the association between body mass index (BMI) and survival, with each epigenetic age acceleration serving as the mediator. Linear models were used to evaluate the association between BMI and its respective mediator, and parametric survival models with a Gompertz distribution and age as the timescale were used as outcome models.

All models were adjusted for sex, educational attainment, smoking status, race/ethnicity, and metabolic health. Metabolically healthy status was defined as the absence of hypertension, hyperglycemia, hypertriglyceridemia and low high-density lipoprotein cholesterol (strict criteria used in "Main model" and all other models except for model "Adjusted for MH using less strict criteria"). Metabolic health with less strict criteria defined metabolically healthy status as: no more than one (≤1) of hypertension, hyperglycemia, hypertriglyceridemia, and low high-density lipoprotein cholesterol. Models additionally adjusted for history of cancer (+ adjusted for history of cancer), lung diseases (+ adjusted for history of lung disease), cardiovascular diseases (+ adjusted for history of CVD) or unhealthy alcohol use (+ adjusted for unhealthy alcohol use) were adjusted for metabolic health using the strict criteria.

The x-axis shows the estimated mean difference in survival time. The left panel shows mean ADE (▲) and mean ACME (●) for low BMI defined as the exposed level set at 19kg/m^2 and the unexposed level set at 27kg/m^2, and the right panel shows the mean ADE and ACME for high BMI defined as the exposed level set at 35kg/m^2 and the unexposed level set at 27kg/m^2. The bars represent the 95% confidence intervals.

Abbreviations: ACME – average causal mediating effects, ADE – average direct effects, BMI – body mass index, MH – metabolic health, HannumAgeAcc – acceleration of age predicted by Hannum epigenetic clocks, PhenoAgeAcc – acceleration of age predicted by PhenoAge epigenetic clocks, GrimAgeAcc – acceleration of age predicted by GrimAge epigenetic clocks, DunedinPACE – rate of ageing in years per chronological year

Supplementary Table 13: Sex stratified, average direct effects and average causally mediating effect of high and low BMI on survival time with each epigenetic age acceleration measure as the mediator

**Exposures**		**High BMI**				**Low BMI**			
**Sex**		**Females**		**Male**		**Females**		**Males**	
**Sample size**		2,234		1,606		2,234		1,606	
**Mediators**	**Effects**	**Est.**	**95% CI**	**Est.**	**95% CI**	**Est.**	**95% CI**	**Est.**	**95% CI**
**HannumAgeAcc**	ADE	-1·23	-3·89,1·62	-2·65	-6·39,1·48	-1·59	-4·85,1·62	-13·65	-20·42,-8·30
	ACME	-0·38	-0·73,-0·12	-0·34	-0·90,0·05	-0·24	-0·64,0·04	-1·14	-2·36,-0·21
	Prop. mediated	0·24		0·11		0·13		0·08	
**PhenoAgeAcc**	ADE	-1·32	-3·99,1·66	-3·24	-6·90,0·79	-1·82	-5·24,1·35	-15·59	-22·56,-10·06
	ACME	-0·28	-0·52,-0·11	-0·03	-0·35,0·30	0·11	-0·11,0·40	-0·48	-1·26,0·12
	Prop. mediated	0·18		0·01		NA		0·03	
**GrimAgeAcc**	ADE	-0·95	-3·75,2·10	-2·41	-6·31,1·61	-2·14	-5·59,1·08	-13·24	-20·37,-7·21
	ACME	-0·98	-1·59,-0·53	-0·21	-0·86,0·37	-0·35	-1·00,0·24	-1·98	-3·76,-0·59
	Prop. mediated	0·51		0·08		0·14		0·13	
**DunedinPACE**	ADE	-1·11	-3·90,1·86	-2·70	-6·63,1·77	-2·15	-5·64,1·09	-15·51	-23·2,-9·53
	ACME	-0·31	-0·56,-0·12	-0·18	-0·54,0·11	0·13	-0·11,0·42	-0·57	-1·44,0·04
	Prop. mediated	0·22		0·06		NA		0·04	

Results from mediation analysis estimating direct effects of high BMI and mediating effects through epigenetic age acceleration measured from epigenetic clocks: HannumAge, PhenoAge GrimAge, and DunedinPACE, stratified by sex. Estimated parameters are expressed as the difference in survival time. The average direct effects and average mediating effects represent the difference in survival time at the exposed level set at a high BMI of 35kg/m^2 compared to the unexposed level set at a BMI of 27kg/m^2. The associations between exposures and mediators were modelled by linear regression. The associations between exposure and mediators with time to mortality were modelled by parametric survival models with Gompertz distribution and chronological age as the underlying timescale. All models were adjusted for age, educational attainment, smoking status, race/ethnicity, and metabolic health.

Abbreviations: ACME – average causal mediating effects, ADE – average direct effects, BMI – body mass index, CI – confidence interval, Est. – estimate, Prop. Mediated – proportion mediated, HannumAgeAcc – acceleration of age predicted by Hannum epigenetic clocks, PhenoAgeAcc – acceleration of age predicted by PhenoAge epigenetic clocks, GrimAgeAcc – acceleration of age predicted by GrimAge epigenetic clocks, DunedinPACE – rate of ageing in years per chronological year

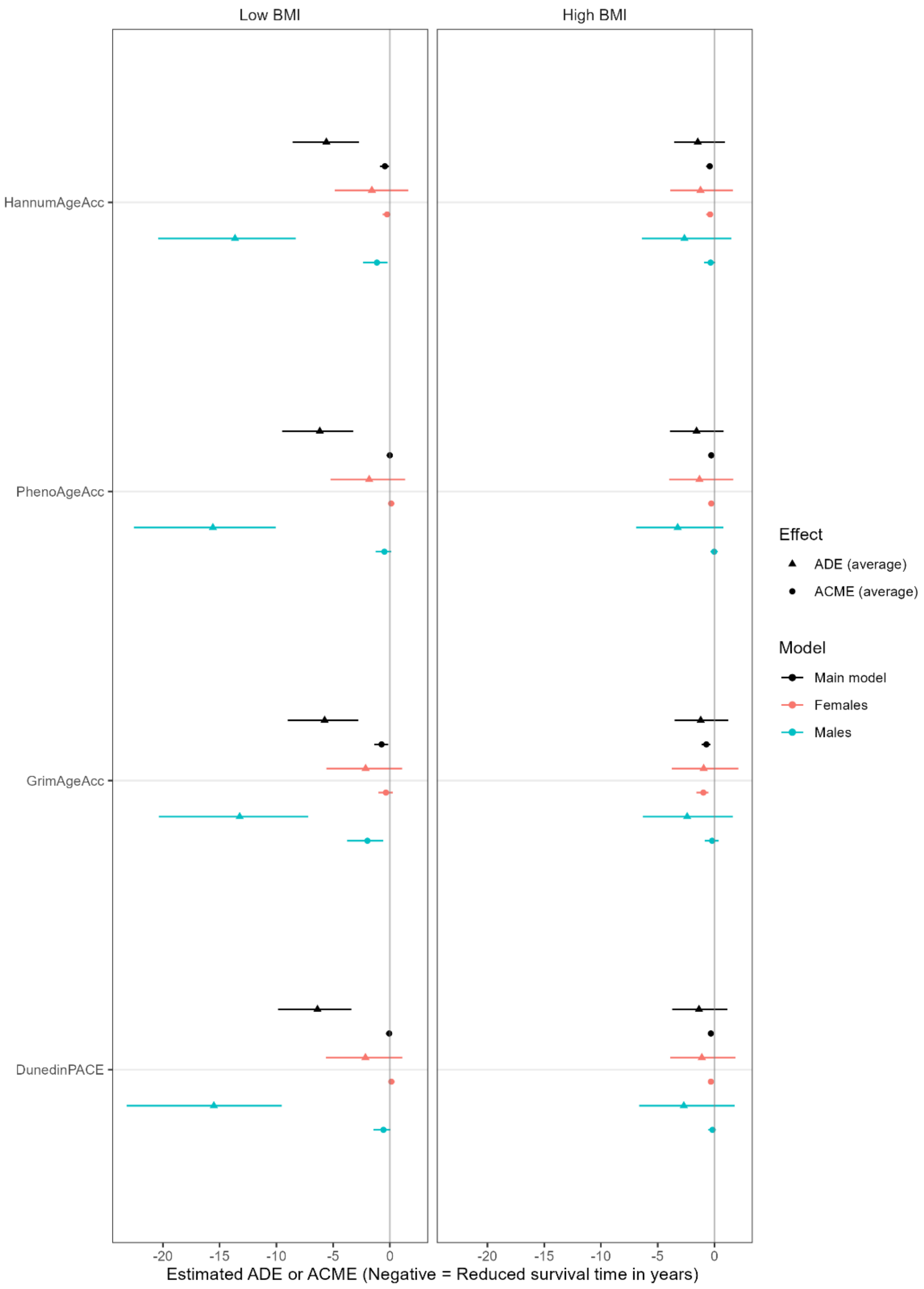

Supplementary Figure 7.

Mediation models of body mass index and survival time by epigenetic age acceleration in females (n = 2,234) and males (n = 1,606).

Average direct effects (ADE) and average causally mediating effects (ACME) were estimated from mediation analyses of the association between BMI and survival, with each epigenetic age acceleration as the mediator. Linear models were used to evaluate the association between BMI and its respective mediator, and parametric survival models with a Gompertz distribution and age as the timescale were used as outcome models. All models were adjusted for age, sex (in Main model, unstratified model), ethnicity/race, smoking status, educational attainment, and metabolic health. Metabolically healthy status used in the main analyses was defined as the absence of hypertension, hyperglycemia, hypertriglyceridemia, and low high-density lipoprotein (strict criteria). The less strict criteria defined metabolically healthy status as the presence of no more than one (≤1) of hypertension, hyperglycemia, hypertriglyceridemia, and low high-density lipoprotein. The x-axis shows the estimated mean difference in survival time. The left panel shows mean ADE (▲) and mean ACME (●) for low BMI defined as the exposed level set at 19kg/m^2 and the unexposed level set at 27kg/m^2, and the right panel shows the mean ADE and ACME for high BMI defined as the exposed level set at 35kg/m^2 and the unexposed level set at 27kg/m^2. The bars represent the 95% confidence intervals.

Abbreviations: ACME – average causal mediating effects, ADE – average direct effects, BMI – body mass index, MH – metabolic health, HannumAgeAcc – acceleration of age predicted by Hannum epigenetic clocks, PhenoAgeAcc – acceleration of age predicted by PhenoAge epigenetic clocks, GrimAgeAcc – acceleration of age predicted by GrimAge epigenetic clocks, DunedinPACE – rate of ageing in years per chronological year

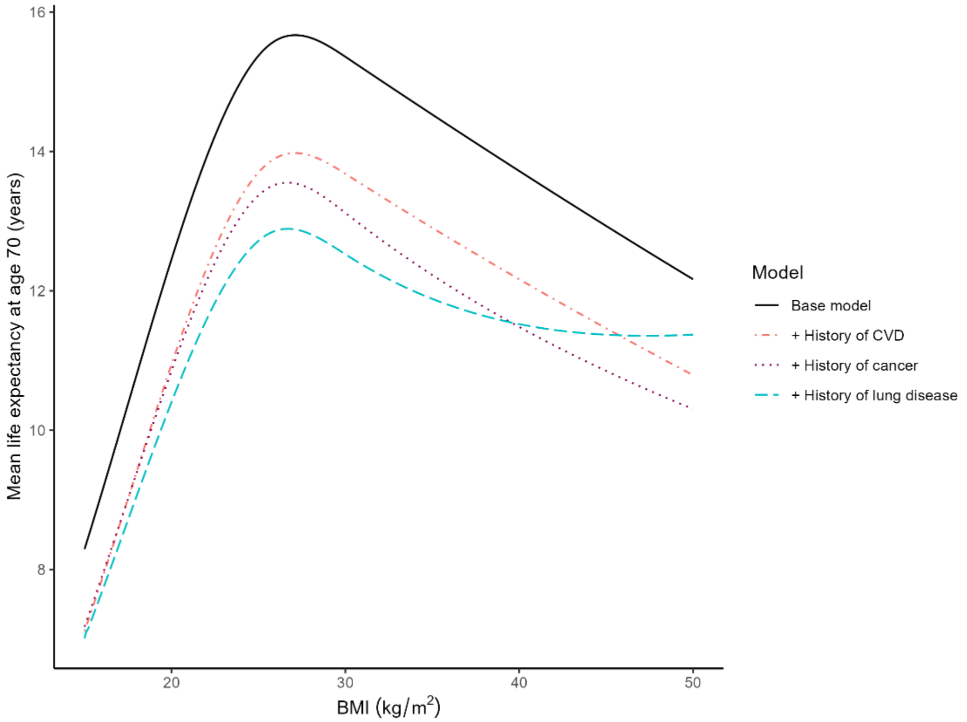

Supplementary Figure 8.

Association between body mass index and mean life expectancy in years. Models were adjusted for age, sex, ethnicity/race, smoking status, educational attainment and metabolic health (Base model, n = 3,840), and further for history of cancer (+ History of cancer, n = 3,837), lung disease (+ History of lung disease, n = 3,839) or cardiovascular disease (+ History of cardiovascular disease, n = 3,836). Mean life expectancies were estimated from the Gompertz proportional hazards model with age as the underlying time scale. The plots were based on predictions made among white males aged 70 years, with mean levels of epigenetic age acceleration measure or BMI, who were never smokers, metabolically unhealthy, and had educational attainment of high school or below. In models further adjusted for disease history, predictions were made among those with a disease history.

Abbreviations: BMI – body mass index, CVD – cardiovascular disease, kg/m^2 – kilograms per square meter,
